# A mixed-methods online survey approach using retrospective self-reporting to characterise congenital ichthyoses across age groups

**DOI:** 10.1186/s13023-026-04358-7

**Published:** 2026-04-18

**Authors:** Talia Elgie, William Davies, Georgina H. Wren

**Affiliations:** 1 School of Psychology, Cardiff University, Tower Building, 70, Park Place, Cardiff, UK; 2 Division of Psychological Medicine and Clinical Neurosciences and Centre for Neuropsychiatric Genetics and Genomics, School of Medicine, Cardiff University, Cardiff, UK; 3 Neuroscience and Mental Health Innovation Institute, Cardiff University, Cardiff, UK

**Keywords:** Ageing, Comorbidity, Genetic counselling, Ichthyosis, Psychodermatology

## Abstract

**Background:**

The ichthyoses are a group of rare, dermatological conditions characterised by dry, thickened scales across the body and impaired skin barrier function. Although ichthyosis may be acquired, the majority of cases are congenital and caused by specific genetic variants. The ichthyoses can cause multiple skin-related issues (e.g. increased risk of infection) and can also have substantial downstream effects on mental health and quality of life; moreover, they are commonly comorbid with a range of medical conditions. Although the symptoms associated with the various ichthyosis subtypes have been reasonably well-defined, how these change over time, and how they compare to one another, has not yet been systematically investigated. Using an online survey approach, we obtained detailed quantitative and qualitative data from adults diagnosed with congenital ichthyosis who self-reported on their skin-related and medical symptoms, and the impact of these, across six time periods; we also obtained data on factors self-reported to contribute to changing health over time, and on participants’ future health concerns.

**Results:**

We recruited a total of 222 adult participants diagnosed with a range of ichthyosis subtypes: ichthyosis vulgaris (*n* = 86), autosomal recessive congenital ichthyosis (*n* = 67), X-linked ichthyosis (*n* = 34), epidermolytic ichthyosis (*n* = 30) and Netherton syndrome (*n* = 8). We showed that the ichthyoses have a lifelong impact on quality of life, particularly for more severe subtypes, but that this diminishes over time and correlates with skin health. Participants with the more severe forms of ichthyosis self-reported adverse effects throughout life on homeostatic health (e.g. ability to maintain body temperature) and in mid-late middle age on mobility and autoimmune health. These participants also reported consistently relatively poor mental health across life, whereas participants with milder forms of ichthyosis reported more variable mental health outcomes across life. Cardiac and metabolic health were generally regarded as being reasonable throughout life across all ichthyosis subtypes. Participants were most concerned about future worsening of their skin condition and associated medical conditions.

**Conclusions:**

Our data provide new information on how ichthyosis subtypes vary, and compare, at different ages. This information is important for identifying optimal intervention points, and for improving genetic counselling processes.

**Supplementary Information:**

The online version contains supplementary material available at 10.1186/s13023-026-04358-7.

## Background

The ichthyoses are a group of rare dermatological conditions, characterised by dry, thickened scales across the body and impaired skin barrier function [1]. Whilst ichthyosis can be acquired (e.g. as a consequence of autoimmune conditions, medication side-effects or nutritional deficiency) [2], most of the > 50 subtypes are congenital and caused by inherited genetic variants [3, 4]. Diagnosis of inherited ichthyosis is usually made at, or shortly after, birth based on the presence of visible skin scaling in combination with consideration of family history, and confirmatory genetic or biochemical testing is often required to classify the exact subtype. Recently, there has been a move towards diagnosing and classifying syndromic and nonsyndromic epidermal differentiation disorders (sEDDs and nEDDs respectively), including the ichthyoses, according to their genetic basis [5, 6]. Although we recognize this innovation, here we use older terminology which is still used and understood (and, in some cases preferred) by many clinicians and patients.

The most common ichthyosis subtype is ichthyosis vulgaris, affecting 1 in 100–250 individuals, caused by loss-of-function variants in the *FLG* (filaggrin) gene (now *FLG*-nEDD). The second most common subtype is X-linked ichthyosis (XLI), which almost exclusively affects males (diagnosed in 1 in 2000–6000 individuals) and results from loss-of-function variants in the X-linked *STS* (steroid sulfatase) gene (now *STS*-sEDD). Rarer subtypes include the autosomal recessive congenital ichthyoses, caused by a variety of genetic changes (ARCI: congenital ichthyosiform erythroderma, lamellar ichthyosis and harlequin ichthyosis), epidermolytic ichthyosis (now *KRT*-nEDD), and Netherton syndrome (now *SPINK5*-sEDD).

Congenital ichthyoses are lifelong conditions with no cure. Skin symptoms can be managed through regular application of a combination of topical treatments designed to restore skin barrier function, minimise water loss and reduce scaling, and/or oral retinoids where appropriate. The severity of skin symptoms can fluctuate with intrinsic factors such as stress, or with external environmental factors (e.g. humidity or temperature) [7].

The characteristic skin phenotype associated with the ichthyoses is linked to several secondary issues: an increased susceptibility to skin infection and pruritis, impairments with sweating and body temperature regulation, a decreased ability to biosynthesise vitamin D, vulnerability to allergies and atopic conditions, conductive hearing impairment due to desquamation in the outer ear, and dry eyes [1]. While some ichthyosis subtypes are nonsyndromic (i.e. solely affecting the skin) [5], others are syndromic i.e. the causal genetic variants result in aberrant protein expression in physiological systems besides the skin, where the gene is typically-expressed [6]. For example, XLI is associated with ophthalmic, neurological, cardiac and reproductive system abnormalities [8].

Living with ichthyosis and its potential consequences (e.g. stigmatisation and bullying) can have adverse effects on affected individuals’ mental health, education and social interactions, and general quality of life [9, 10]. Additionally, although life expectancy is generally normal in individuals with ichthyosis, comorbidities such as arrhythmias recently reported in XLI, might predispose to chronic and serious health conditions [11].

While the (extra)cutaneous features and impacts of the various ichthyosis subtypes have previously been comprehensively described and compared to unaffected individuals, how these change throughout life and how they compare across the subtypes has not yet been systematically investigated. The concerns of individuals with ichthyosis regarding support for management of their condition in the future have also not received much research attention to date. Obtaining data relating to the course of the ichthyoses, and documenting affected individuals’ worries, has been identified as a priority for research by patient support groups, and will be important in terms of highlighting optimal intervention points for advice and treatments, and for providing genetic counselling regarding likely course of the conditions.

To address these knowledge gaps, we developed a comprehensive online survey, to be distributed worldwide, through which individuals affected by congenital ichthyosis could retrospectively self-report their health-related experiences throughout their life and future concerns in both a quantitative and a qualitative manner. The nature and content of our survey were informed by discussions with a focus group comprising 11 members of the Ichthyosis Support Group UK diagnosed with seven different subtypes of ichthyosis [12] and with participants in social media patient support groups. Online surveys have numerous strengths, particularly in terms of the understanding of rare diseases [13]: large, diverse samples can be recruited rapidly, individuals can choose when and where to complete the survey and can report their own perception of their condition and its effects (rather than this being vicariously reported by clinicians), and, if data are collected anonymously, participants may be more willing to discuss sensitive matters.

## Methods

### Participant recruitment

The survey URL was advertised as widely as possible around the world via relevant charities, social media and associated patient support groups, and to previous participants in our studies in this area who had consented to be recontacted. Inclusion criteria were that participants needed to be adults (> 18yrs of age) and to have received a confirmed clinical diagnosis of an ichthyosis subtype (ideally based on genetic testing); there were no exclusion criteria. Participants did not receive any form of reimbursement.

### Survey description

The survey was created in Qualtrics [14], was presented solely in English, and allowed for anonymous completion in ~ 30–40 minutes. An initial set of demographic questions asked participants about their age, their clinical diagnosis (i.e. subtype of ichthyosis), their country of residence, their ethnic group, and their sex assigned at birth. Participants were then asked the general opening question: ‘To what extent would you say that ichthyosis has impacted your life at the following time periods?’: Childhood (0-12yrs), Adolescence (12-18yrs), Young Adulthood (18-30yrs), Young-Mid Adulthood (30-40yrs), Mid Adulthood (40-50yrs) and Mid-Older Adulthood (> 50yrs). Response options ranged from 1=’not at all’ to 5=’extremely’. Participants were subsequently asked to rate the severity of their skin condition (scaling and redness) at the aforementioned time periods from 1=’mild’ to 4=’extremely severe’ relative to exemplar clinical images from Congenital Ichthyosis Severity Index [15]; if their skin condition had changed throughout life, they were also asked to highlight any contributory factors from the following list: change in personal care (e.g. improved bathing or skincare routine), change in personal circumstances (e.g., lifestyle changes, significant life events, social support), change in living conditions (e.g., moving to an area with a different climate), change in medication or treatments (e.g., new prescription or dosage), no obvious cause, changes in medical or scientific advice (e.g., new treatment guidelines, improved medical guidance), or other. For both initial questions, participants could provide qualitative feedback to support their responses. Next, respondents were invited to complete eight separate subsections about aspects of their health known to be related to ichthyosis (bone health, cardiovascular health (e.g. blood pressure, heart problems, circulation), autoimmune health (e.g. arthritis, type I diabetes), metabolic health (e.g. type 2 diabetes, thyroid conditions, cholesterol levels), psychological/mental health, homeostatic health (e.g. ability to maintain a constant body temperature or fluid balance), mobility, and social interactions. For each of the time periods above, participants rated their health on a scale from 1=’very poor’ to 5=’very good’; they were also invited to self-report which factors contributed to any changes in health over time, and to provide additional qualitative feedback. Participants’ perceptions of healthcare provision over time were then assessed, but these data are not analysed or reported here. Finally, respondents were asked to report on any prospective concerns they had in the following areas on a scale from 1=’not at all concerned’ to 5=’extremely concerned’: worsening of skin, worsening of other medical or psychological conditions, inability of relatives/carers to help with skincare, access to sufficient healthcare provision, and issues related to menopause/reproductive health. Again, qualitative feedback in these areas was solicited.

### Quantitative analysis and statistics

Data are presented as proportions, or as mean±standard error of the mean, for all ichthyosis subtypes combined, and for each individual subtype. For symptom changes over time, data are presented separately for participants > 50yrs of age who self-reported at all time periods assessed, and for participants who self-reported for any time period. Data reported for multiple time periods by individuals were analysed by Friedman Test followed by pairwise comparisons by Wilcoxon Signed Ranks Test Bonferroni-corrected for 15 possible comparisons (i.e. p-values < 0.0033 were regarded as significant and are indicated by letters in Tables). Between-condition symptom scores were compared using Kruskal-Wallis H-test at each time period using all participants’ scores, with p-values < 0.0083 regarded as significant after Bonferroni-correction to account for testing at six time periods; pairwise comparisons with p-values < 0.005 (Bonferroni-corrected for 10 comparisons) were regarded as significant and are indicated by Arabic numerals in Tables. Categorical data were analysed by chi-squared test with Bonferroni correction applied to account for multiple testing where required as above; standardised residuals <-1.96 or > 1.96 were used to identify cells differing significantly (*p* < 0.05) from expected values. Quantitative data are available in Additional File 1.

### Qualitative analysis

Qualitative responses from the free text boxes were analysed to support significant findings from the quantitative analysis using a mix of deductive and inductive content analysis according to previously-published guidelines [16, 17]. Responses for each section were published separately. A content map summarising participants’ qualitative responses can be found in Additional File 2.

## Results

### Participant characteristics

In total, 275 individuals initially responded to our survey. Data from individuals who did not complete > 90% of the survey, who did not self-report their subtype of ichthyosis (correct self-reporting of diagnostic subtype can be challenging), who reported a diagnosis of acquired (as opposed to congenital) ichthyosis, and from individuals diagnosed with conditions for which there were fewer than seven responses were not analysed further here.

The characteristics of the remaining 222 participants are reported in Table 1. Most participants were middle-aged (40-50yrs), resided in United Kingdom or United States of America, and were of White ethnicity. 38% had been diagnosed with ichthyosis vulgaris, 30% with a form of autosomal recessive congenital ichthyosis (ARCI), 15% with X-linked ichthyosis (XLI), 13% with epidermolytic ichthyosis (EI) and 4% with Netherton syndrome. As expected, respondents diagnosed with XLI were all male; the majority of respondents for the other ichthyosis subtypes (> 67%) were assigned female at birth.

**Table 1:** Sample demographics

Type of ichthyosis	Subtype of ichthyosis	Number of participants	Sex assigned at birth (male; female)	Participant age (yrs) (mean±SEM§)	Country of residence (United Kingdom; United States of America; Elsewhere/No response	Ethnicity (White; other)
Ichthyosis vulgaris	-	83 (37%)	27 (33%);56 (67%)	51.5 ± 1.7	41 (49%);14 (17%); 28 (33%)	65 (78%);18 (22%)
Autosomal recessive congenital ichthyosis (ARCI)(*n* = 67)	Lamellar ichthyosis	41 (18%)	10 (24%);31 (76%))	43.0 ± 2.2	9 (22%);12 (29%); 20 (49%)	31 (76%);10 (24%)
	Congenital Ichthyosiform Erythroderma (CIE)	24 (11%)	6 (25%);18 (75%)	46.5 ± 2.9	9 (38%);9 (38%); 6 (24%)	20 (83%);4 (17%)
	Harlequin ichthyosis	2 (1%)	0 (0%);2 (100%)	50.0 ± 15.0	2 (100%);0 (0%); 0 (0%)	2 (100%);0 (0%)
X-linked ichthyosis	-	34 (15%)	34 (100%);0 (0%)	54.3 ± 2.6	19 (56%);6 (18%); 9 (26%)	31 (91%);3 (9%)
Epidermolytic ichthyosis	-	30 (13%)	9 (30%);21 (70%)	45.2 ± 2.6	20 (67%);7 (23%);3 (10%)	30 (100%);0 (0%)
Netherton syndrome	-	8 (4%)	1 (13%);7 (87%)	41.6 ± 3.1	2 (25%);3 (38%);3 (38%)	7 (88%);1 (12%)

§ Standard error of the mean

### General impact of ichthyosis on quality of life at different time periods

#### Quantitative analysis

Participants reported a substantial impact of ichthyosis on their quality of life (mean scores of ≥ 3.5/5 for almost all subtypes and time periods assessed); the largest effects were reported for ARCI, EI and Netherton syndrome during childhood (< 18yrs) (Table 2). Across the overall sample, and across most ichthyosis subtypes, the impact of the condition on individuals’ lives varied significantly by developmental time period, with a general trend towards reduced impact with increasing age; for EI, whilst this result was non-significant, the pattern of responses followed the same general trend (Table 2). Comparing across ichthyosis types at each time period for this measure indicated significant between-group effects for childhood (0-12yrs) and adolescence (12-18yrs), but not for later time periods. Follow-up pairwise comparison indicated that between 0-12yrs, individuals diagnosed with XLI self-reported a significantly lower impact on life score than individuals with EI, and between 12-18yrs, individuals with XLI self-reported a significantly lower impact on life score than individuals diagnosed with EI or ARCI.

**Table 2:** Impact on quality of life across time periods

Type of ichthyosis	Sample	Self-reported impact on quality of life at various life stages (mean±SEM§) (1 = no impact at all, 5 = extreme impact)						Statistical analysis of change over time
		0-12yrs	12-18yrs	18-30yrs	30-40yrs	40-50yrs	> 50yrs	
All types combined	Only participants self-reporting for all timepoints (*n* = 102)	4.25 ± 0.11^a, b^	4.35 ± 0.08^c, d,e, f^	4.08 ± 0.08^c, g,h^	3.76 ± 0.10^a, d,g^	3.77 ± 0.10^b, e,h^	3.87 ± 0.11^f^	χ^2^[5] = 81.4, *p* < 0.001*
	Participants self-reporting for any timepoint (*n* = 102–222)	4.49 ± 0.06	4.44 ± 0.05	4.04 ± 0.06	3.88 ± 0.07	3.83 ± 0.08	3.87 ± 0.11	-
Ichthyosis vulgaris	Only participants self-reporting for all timepoints (*n* = 46)	4.15 ± 0.15	4.35 ± 0.10^a^	4.07 ± 0.12^b^	3.76 ± 0.13^a, b^	3.85 ± 0.13	4.00 ± 0.15	χ^2^[5] = 27.2, *p* < 0.001*
	Participants self-reporting for any timepoint (*n* = 46–83)	4.25 ± 0.11	4.46 ± 0.08	4.07 ± 0.09	3.80 ± 0.10	3.83 ± 0.11	4.00 ± 0.15	-
Autosomal recessive congenital ichthyosis (ARCI)	Only participants self-reporting for all timepoints (*n* = 23)	4.52 ± 0.23	4.61 ± 0.12^a^	4.22 ± 0.22	4.04 ± 0.21	3.89 ± 0.24^a^	4.00 ± 0.24	χ^2^[5] = 28.9, *p* < 0.001*
	Participants self-reporting for any timepoint (*n* = 23–67)	4.57 ± 0.09	^2^4.61 ± 0.08	4.06 ± 0.10	4.06 ± 0.12	3.99 ± 0.18	4.00 ± 0.24	-
X-linked ichthyosis	Only participants self-reporting for all timepoints (*n* = 21)	3.86 ± 0.25	3.90 ± 0.23	3.86 ± 0.19	3.48 ± 0.19	3.48 ± 0.21	3.48 ± 0.22	χ^2^[5] = 16.8, *p* = 0.005*
	Participants self-reporting for any timepoint (*n* = 21–34)	^1^4.03 ± 0.20	^1,2^3.88 ± 0.19	3.79 ± 0.17	3.52 ± 0.17	3.54 ± 0.20	3.48 ± 0.22	-
Epidermolytic ichthyosis	Only participants self-reporting for all timepoints (*n* = 11)	4.73 ± 0.27	4.64 ± 0.15	4.27 ± 0.14	3.73 ± 0.45	3.73 ± 0.45	3.73 ± 0.41	χ^2^[5] = 14.5, *p* = 0.013
	Participants self-reporting for any timepoint (*n* = 11–30)	^1^4.70 ± 0.14	^1^4.60 ± 0.10	4.23 ± 0.15	4.04 ± 0.24	3.95 ± 0.28	3.73 ± 0.41	-
Netherton syndrome	Only participants self-reporting for all timepoints (*n* = 1)	5.00 ± 0.00	5.00 ± 0.00	4.00 ± 0.00	4.00 ± 0.00	4.00 ± 0.00	5.00 ± 0.00	-
	Participants self-reporting for any timepoint (*n* = 1–8)	4.75 ± 0.16	4.50 ± 0.27	4.00 ± 0.38	4.43 ± 0.30	3.50 ± 0.50	5.00 ± 0.00	-
**Statistical analysis of between-group effects**	-	H[4] = 15.1, *p* = 0.004*	H[4] = 14.7, *p* = 0.005*	H[4] = 3.7, *p* = 0.44	H[4] = 12.4, *p* = 0.012	H[4] = 5.8, *p* = 0.22	H[4] = 6.0, *p* = 0.20	-

§ Standard error of the mean. Where individuals self-reported across all time periods, data were analysed by Friedman test (Bonferroni-corrected significant p-values highlighted by asterisks); significant Bonferroni-corrected pairwise comparisons by Wilcoxon Signed Ranks test are highlighted by the same superscript letters within a row. Between-group comparisons were conducted using Kruskal-Wallis H-test (Bonferroni-corrected significant p-values highlighted by asterisks); Bonferroni-corrected significant pairwise comparisons are highlighted by the same superscript Arabic numerals within a column

#### Qualitative analysis

Participants clearly described the substantial impact of their condition throughout life, including negative effects on self-esteem and socialising, and the constant need to ‘manage’ their skin. Reduced impacts of the condition on quality of life over time were frequently attributed to increased personal acceptance (leading to greater self-confidence), as well as advances in treatment and more consistent symptom management. The experiences of individuals with XLI, compared to ARCI or EI, between 12-18yrs, vary greatly within the free-text responses, and, as such, the exact reasons for the differential impact scores for these conditions at this time period are hard to establish. However, some individuals with ARCI reported that access to the symptom-alleviating retinoid medication acitretin was only available from late-adolescence (> 16yrs) onwards.

### Skin condition at different time periods

#### Quantitative analysis

For the overall sample, and for most types of ichthyosis, skin condition appeared to be related to time period, with a general reduction in severity with increasing participant age. XLI appeared to be an exception to this, with self-reported skin condition remaining comparatively stable over the various time periods assessed (Table 3). At the youngest time period assessed (0-12yrs), participants with some ichthyosis subtypes (ARCI, EI and Netherton syndrome) self-reported a severe-extremely severe skin condition (mean scores of 3–4). For other time periods and conditions, skin severity was generally rated as being between moderate-severe (mean scores of 2–3)(Table 3). Comparing across ichthyosis subtypes at each time period for this measure indicated significant between-group effects for the ‘early childhood’ (0-12yrs) time period only. Follow-up pairwise comparisons indicated that, between 0-12yrs of age specifically, self-reported skin severity in ARCI, EI and Netherton syndrome is significantly worse than in XLI and IV.

**Table 3:** Skin condition across time periods

Type of ichthyosis	Sample	Self-reported skin condition at various life stages (mean±SEM§) (1 = mild, 4 = extremely severe)						Statistical significance of change over time
		0-12yrs	12-18yrs	18-30yrs	30-40yrs	40-50yrs	> 50yrs	
All types combined	Only participants self-reporting for all timepoints (*n* = 104)	2.80 ± 0.09^a, b,c, d^	2.74 ± 0.09^e, f,g, h^	2.43 ± 0.08^a, e^	2.31 ± 0.07^b, f^	2.33 ± 0.07^c, g^	2.37 ± 0.08^d, h^	χ^2^[5] = 70.4, *p* < 0.001*
	Participants self-reporting for any timepoint (*n* = 104–214)	2.89 ± 0.06	2.70 ± 0.06	2.42 ± 0.06	2.29 ± 0.05	2.30 ± 0.06	2.37 ± 0.08	-
Ichthyosis vulgaris	Only participants self-reporting for all timepoints (*n* = 44)	2.59 ± 0.14	2.70 ± 0.14^a^	2.45 ± 0.12	2.25 ± 0.10^a^	2.27 ± 0.19	2.30 ± 0.11	χ^2^[5] = 20.1, *p* < 0.001*
	Participants self-reporting for any timepoint (*n* = 44–79)	^4,5,6^2.67 ± 0.11	2.70 ± 0.09	2.48 ± 0.09	2.21 ± 0.08	2.19 ± 0.08	2.30 ± 0.11	-
Autosomal recessive congenital ichthyosis (ARCI)	Only participants self-reporting for all timepoints (*n* = 25)	3.32 ± 0.17^a, b,c, d^	3.12 ± 0.17^e, f,g, h^	2.64 ± 0.18^a, e^	2.56 ± 0.18^b, f^	2.48 ± 0.18^c, g^	2.56 ± 0.19^d, h^	χ^2^[5] = 48.8, *p* < 0.001*
	Participants self-reporting for any timepoint (*n* = 25–67)	^1,4^3.13 ± 0.10	2.79 ± 0.11	2.39 ± 0.11	2.38 ± 0.12	2.46 ± 0.13	2.56 ± 0.19	-
X-linked ichthyosis	Only participants self-reporting for all timepoints (*n* = 22)	2.36 ± 0.20	2.32 ± 0.18	2.09 ± 0.15	2.14 ± 0.15	2.18 ± 0.16	2.23 ± 0.16	χ^2^[5] = 3.9, *p* = 0.57
	Participants self-reporting for any timepoint (*n* = 22–34)	^1,2,3^2.44 ± 0.15	2.41 ± 0.13	2.15 ± 0.11	2.19 ± 0.13	2.21 ± 0.13	2.23 ± 0.16	-
Epidermolytic ichthyosis	Only participants self-reporting for all timepoints (*n* = 12)	3.17 ± 0.24	2.75 ± 0.22	2.50 ± 0.15	2.25 ± 0.13	2.42 ± 0.15	2.33 ± 0.19	χ^2^[5] = 17.4, *p* = 0.004*
	Participants self-reporting for any timepoint (*n* = 12–27)	^2,5^3.26 ± 0.15	2.81 ± 0.14	2.63 ± 0.12	2.42 ± 0.12	2.63 ± 0.16	2.33 ± 0.19	-
Netherton syndrome	Only participants self-reporting for all timepoints (*n* = 1)	4.00 ± 0.00	4.00 ± 0.00	3.00 ± 0.00	3.00 ± 0.00	3.00 ± 0.00	4.00 ± 0.00	-
	Participants self-reporting for any timepoint (*n* = 1–7)	^3,6^3.71 ± 0.18	2.86 ± 0.34	2.43 ± 0.37	2.86 ± 0.34	2.33 ± 0.33	4.00 ± 0.00	-
**Statistical analysis of between-group effects**	-	H[4] = 27.5, *p* < 0.001*	H[4] = 5.6, *p* = 0.23	H[4] = 6.2, *p* = 0.18	H[4] = 7.6, *p* = 0.11	H[4] = 6.2, *p* = 0.19	H[4] = 4.8, *p* = 0.31	-

§ Standard error of the mean. Where individuals self-reported across all time periods, data were analysed by Friedman test (Bonferroni-corrected significant p-values highlighted by asterisks); significant Bonferroni-corrected pairwise comparisons by Wilcoxon Signed Ranks test are highlighted by the same superscript letters within a row. Between-group comparisons were conducted using Kruskal-Wallis H-test (Bonferroni-corrected significant p-values highlighted by asterisks); Bonferroni-corrected significant pairwise comparisons are highlighted by the same superscript Arabic numerals within a column

Amongst participants from the overall sample who reported that their skin condition changed significantly over time, ~ 60% attributed this change to differences in self-care and ~ 50% to changes in medications/treatments; the remaining factors were less frequently reported (< 30%)(Table 4). The only contributory factor that differed significantly with respect to changes in skin condition across the ichthyosis subtypes was ‘change in medication or treatments’. This significant result was driven by a higher than expected number of affirmative responses (100%) by individuals diagnosed with Netherton syndrome (Table 4).

**Table 4:** Factors contributing to changes in skin condition across time periods

Type of ichthyosis	Number of participants reporting changing skin condition	Number (%) of participants reporting factor as contributory towards changing skin condition§						
		Change in self-care	Change in personal circumstances	Change in living conditions	Change in medication or treatments	No obvious cause	Changes in medical or scientific advice	Other
All types combined	203	123 (61%)	54 (27%)	53 (26%)	96 (47%)	49 (24%)	34 (17%)	29 (14%)
Ichthyosis vulgaris	76	45 (59%)	15 (20%)	19 (25%)	32 (42%)	19 (25%)	11 (15%)	13 (17%)
Autosomal recessive congenital ichthyosis (ARCI)	65	44 (68%)	26 (40%)	20 (31%)	36 (55%)	9 (14%)	13 (20%)	5 (8%)
X-linked ichthyosis	29	13 (45%)	3 (10%)	6 (21%)	7 (24%)	9 (31%)	3 (10%)	4 (14%)s
Epidermolytic ichthyosis	26	17 (65%)	7 (27%)	5 (19%)	16 (62%)	10 (39%)	6 (23%)	5 (19%)
Netherton syndrome	7	4 (57%)	3 (43%)	3 (43%)	7 (100%)	2 (29%)	1 (14%)	2 (29%)
**Statistical analysis of between-group effects**	-	χ^2^[4] = 4.7, *p* = 0.32	χ^2^[4] = 12.7, *p* = 0.013	χ^2^[4] = 2.9, *p* = 0.58	χ^2^[4] = 18.6, *p* = 0.001*	χ^2^[4] = 7.5, *p* = 0.11	χ^2^[4] = 2.4, *p* = 0.66	χ^2^[4] = 4.5, *p* = 0.34

§ Between-group comparisons were conducted using chi-squared test, and significant Bonferroni-corrected p-values are highlighted with asterisks

#### Qualitative analysis

Reduced skin severity with increasing age across the overall sample was frequently linked to the positive effects of treatments, as well as to improved self-management of the skin. More specifically, the severe impact of ARCI and EI during childhood and adolescence was attributed to late access to effective treatments such as acitretin and etretinate, and the tendency of these conditions to resolve with age. In comparison, individuals with XLI described their skin condition to be relatively consistent across the time periods assessed, with any improvements attributed to lifestyle changes such as moving to a warmer climate. Individuals with Netherton syndrome detailed significant side-effects to certain medications including skin thinning, thus prompting a change in treatment.

### Bone health at different time periods

#### Quantitative analysis

As expected, bone health scores across all subtypes of ichthyosis tended to be worse (i.e. decrease) at more advanced ages (Table 5). Mean scores were particularly low (2–3, indicating poor-fair health) in the oldest participants (> 50yrs) diagnosed with ARCI, and in the small sample of older participants diagnosed with Netherton syndrome (> 30yrs). We found evidence for significant between-group effects from the age of 30yrs onwards, with the most consistent subtype differences being poorer self-reported bone health in participants diagnosed with ARCI compared to participants with either IV or XLI. For participants self-reporting changes in bone health over time, ‘no obvious cause’ was the most commonly endorsed response option (~ 35%)(Additional File 3). There were no significant between-group differences in terms of factors identified as being contributors to changing bone health.

**Table 5:** Bone health across time periods

Type of ichthyosis	Sample	Self-reported bone health at various life stages (mean±SEM§) (1 = very poor, 5 = very good)						Statistical analysis of change over time
		0-12yrs	12-18yrs	18-30yrs	30-40yrs	40-50yrs	> 50yrs	
All types combined	Only participants self-reporting for all timepoints (*n* = 73)	4.42 ± 0.09^a, b,c^	4.42 ± 0.08^d, e,f^	4.36 ± 0.09^g, h,i^	4.14 ± 0.12^a, d,g, j,k^	3.84 ± 0.14^b, e,h, j,l^	3.58 ± 0.16^c, f,i, k,l^	χ^2^[5] = 121.0, *p* < 0.001*
	Participants self-reporting for any timepoint (*n* = 85–174)	4.35 ± 0.06	4.38 ± 0.06	4.17 ± 0.07	3.92 ± 0.09	3.69 ± 0.11	3.40 ± 0.15	-
Ichthyosis vulgaris	Only participants self-reporting for all timepoints (*n* = 29)	4.66 ± 0.10	4.69 ± 0.09	4.69 ± 0.09	4.66 ± 0.10	4.48 ± 0.14	4.28 ± 0.18	χ^2^[5] = 31.1, *p* < 0.001*
	Participants self-reporting for any timepoint (*n* = 36–62)	4.55 ± 0.08	4.52 ± 0.09	4.28 ± 0.13	^1,3^4.23 ± 0.14	^1,3^4.18 ± 0.14	^1^3.86 ± 0.21	-
Autosomal recessive congenital ichthyosis (ARCI)	Only participants self-reporting for all timepoints (*n* = 17)	3.88 ± 0.28	3.82 ± 0.21^a^	3.59 ± 0.26^b^	3.29 ± 0.28	2.94 ± 0.30	2.53 ± 0.26^a, b^	χ^2^[5] = 37.4, *p* < 0.001*
	Participants self-reporting for any timepoint (*n* = 20–56)	4.13 ± 0.14	4.16 ± 0.12	3.91 ± 0.13	^3,4^3.50 ± 0.18	^2,3^3.10 ± 0.22	^1^2.60 ± 0.27	-
X-linked ichthyosis	Only participants self-reporting for all timepoints (*n* = 15)	4.53 ± 0.17	4.53 ± 0.17	4.53 ± 0.17	4.40 ± 0.24	4.07 ± 0.28	3.73 ± 0.36	χ^2^[5] = 27.8, *p* < 0.001*
	Participants self-reporting for any timepoint (*n* = 16–28)	4.46 ± 0.16	4.56 ± 0.12	4.50 ± 0.13	^2,4^4.38 ± 0.17	^2^4.09 ± 0.24	3.81 ± 0.34	-
Epidermolytic ichthyosis	Only participants self-reporting for all timepoints (*n* = 12)	4.50 ± 0.15	4.50 ± 0.15	4.42 ± 0.15	3.75 ± 0.25	3.25 ± 0.37	3.17 ± 0.41	χ^2^[5] = 29.5, *p* < 0.001*
	Participants self-reporting for any timepoint (*n* = 12–24)	4.38 ± 0.15	4.38 ± 0.15	4.22 ± 0.14	3.81 ± 0.19	^1^3.06 ± 0.31	3.17 ± 0.41	-
Netherton syndrome	Only participants self-reporting for all timepoints (*n* = 0)	-	-	-	-	-	-	-
	Participants self-reporting for any timepoint (*n* = 1–7)	3.60 ± 0.40	4.20 ± 0.37	3.60 ± 0.40	^1,2^2.57 ± 0.30	3.00 ± 1.00	2.00 ± 0.00	-
**Statistical analysis of between-group effects**	-	H[4] = 10.3, *p* = 0.036	H[4] = 7.5, *p* = 0.11	H[4] = 12.5, *p* = 0.014	H[4] = 27.8, *p* < 0.001*	H[4] = 21.6, *p* < 0.001*	H[4] = 13.9, *p* = 0.008*	-

§ Standard error of the mean. Where individuals self-reported across all time periods, data were analysed by Friedman test (Bonferroni-corrected significant p-values highlighted by asterisks); significant Bonferroni-corrected pairwise comparisons by Wilcoxon Signed Ranks test are highlighted by the same superscript letters within a row. Between-group comparisons were conducted using Kruskal-Wallis H-test (Bonferroni-corrected significant p-values highlighted by asterisks); Bonferroni-corrected significant pairwise comparisons are highlighted by the same superscript Arabic numerals within a column

#### Qualitative analysis

Most participants attributed increasing joint pain and stiffness to normal ageing processes. However, many also reported early-onset arthritis, which they linked to their specific ichthyosis subtype, alongside worsening bone conditions (such as increased fracture risk) due to prolonged steroid use. Individuals with ARCI consistently expressed concerns about bone health and pain from 30 years of age onwards, describing significant impacts on their independence and ability to participate in activities such as sports. While individuals with IV did not rate their bone health poorly in the quantitative measures, some revealed issues in their free-text responses, including osteoporosis and increased fracture susceptibility, though the direct relationship to their ichthyosis subtype remained unclear. In contrast, very few individuals with XLI reported any bone health-related concerns.

### Cardiovascular health at different time periods

#### Quantitative analysis

Again, as expected, cardiovascular health scores across all subtypes of ichthyosis tended to be worse at more advanced ages (Table 6). Mean scores across all subtypes and all time periods were > 3/5, indicating fair-very good ratings with respect to this aspect of health over time. We did not identify any significant between-group differences at any time period with respect to cardiovascular health. For participants self-reporting changes in cardiovascular health over time, ‘no obvious cause’ was the most commonly-endorsed response option (~ 35%)(Additional File 4). There were no significant between-group differences in terms of factors identified as being contributors to changing cardiovascular health.

**Table 6:** Cardiovascular health across time periods

Type of ichthyosis	Sample	Self-reported cardiovascular health at various life stages (mean±SEM§) (1 = very poor, 5 = very good)						Statistical analysis of change over time
		0-12yrs	12-18yrs	18-30yrs	30-40yrs	40-50yrs	> 50yrs	
All types combined	Only participants self-reporting for all timepoints (*n* = 84)	4.46 ± 0.08^a, b,c, d^	4.43 ± 0.08^e, f,g^	4.32 ± 0.09^a, h,I, j^	4.13 ± 0.10^b, e,h, k,l^	3.82 ± 0.12^c, f,I, k^	3.63 ± 0.12^d, g,j, l^	χ^2^[5] = 148.0, *p* < 0.001*
	Participants self-reporting for any timepoint (*n* = 96–194)	4.42 ± 0.06	4.38 ± 0.05	4.21 ± 0.06	3.97 ± 0.07	3.73 ± 0.09	3.66 ± 0.11	-
Ichthyosis vulgaris	Only participants self-reporting for all timepoints (*n* = 34)	4.41 ± 0.13^a^	4.41 ± 0.13^b^	4.35 ± 0.13^c, d^	4.18 ± 0.15^e^	3.85 ± 0.18^c, e^	3.71 ± 0.19^a, b,d^	χ^2^[5] = 48.7, *p* < 0.001*
	Participants self-reporting for any timepoint (*n* = 42–72)	4.48 ± 0.09	4.45 ± 0.09	4.31 ± 0.10	4.08 ± 0.11	3.83 ± 0.14	3.74 ± 0.17	-
Autosomal recessive congenital ichthyosis (ARCI)	Only participants self-reporting for all timepoints (*n* = 22)	4.36 ± 0.20^a^	4.32 ± 0.20^b^	4.18 ± 0.20^c^	3.95 ± 0.24^d^	3.68 ± 0.30	3.50 ± 0.28^a, b,c, d^	χ^2^[5] = 42.6, *p* < 0.001*
	Participants self-reporting for any timepoint (*n* = 24–62)	4.32 ± 0.11	4.28 ± 0.11	4.06 ± 0.11	3.80 ± 0.16	3.57 ± 0.22	3.58 ± 0.26	-
X-linked ichthyosis	Only participants self-reporting for all timepoints (*n* = 17)	4.65 ± 0.12^a^	4.53 ± 0.12	4.35 ± 0.17	4.24 ± 0.16	3.88 ± 0.24	3.59 ± 0.27^a^	χ^2^[5] = 35.2, *p* < 0.001*
	Participants self-reporting for any timepoint (*n* = 19–30)	4.52 ± 0.14	4.45 ± 0.14	4.23 ± 0.14	4.14 ± 0.15	3.88 ± 0.19	3.53 ± 0.25	-
Epidermolytic ichthyosis	Only participants self-reporting for all timepoints (*n* = 11)	4.55 ± 0.16	4.55 ± 0.16	4.45 ± 0.21	4.18 ± 0.23	3.91 ± 0.25	3.73 ± 0.33	χ^2^[5] = 24.5, *p* < 0.001*
	Participants self-reporting for any timepoint (*n* = 11–24)	4.45 ± 0.11	4.46 ± 0.10	4.33 ± 0.12	4.10 ± 0.14	3.72 ± 0.18	3.73 ± 0.33	-
Netherton syndrome	Only participants self-reporting for all timepoints (*n* = 0)	-	-	-	-	-	-	-
	Participants self-reporting for any timepoint (*n* = 0–7)	4.00 ± 0.26	4.00 ± 0.31	3.83 ± 0.31	3.00 ± 0.37	3.00 ± 0.00	-	-
**Statistical analysis of between-group effects**	-	H[4] = 4.8, *p* = 0.30	H[4] = 3.7, *p* = 0.44	H[4] = 5.4, *p* = 0.25	H[4] = 8.2, *p* = 0.086	H[4] = 3.1, *p* = 0.54	H[3] = 0.6, *p* = 0.91	-

§ Standard error of the mean. Where individuals self-reported across all time periods, data were analysed by Friedman test (Bonferroni-corrected significant p-values highlighted by asterisks); significant Bonferroni-corrected pairwise comparisons by Wilcoxon Signed Ranks test are highlighted by the same superscript letters within a row. Between-group comparisons were conducted using Kruskal-Wallis H-test (Bonferroni-corrected significant p-values highlighted by asterisks); Bonferroni-corrected significant pairwise comparisons are highlighted by the same superscript Arabic numerals within a column

#### Qualitative analysis

< 28% of participants provided qualitative information about their cardiovascular health, implying that many individuals were not experiencing any notable issues. For those who provided information, concerns largely centred around hypertension (usually successfully managed by medication), or ventricular tachycardias such as atrial fibrillation, usually developing in mid-adulthood.

### Autoimmune health at different time periods

#### Quantitative analysis

The pattern of data for autoimmune health was similar to that seen for cardiovascular health. Scores across all subtypes of ichthyosis tended to be worse (i.e. decrease) at more advanced ages (Table 7). The majority of mean scores across subtypes and time periods were > 3/5, indicating generally fair-very good ratings with respect to this aspect of health over time. Mean scores between 2 and 3 (indicative of poor-fair health) were reported for participants diagnosed with ARCI (> 50yrs), with EI (> 40yrs) and Netherton syndrome (> 50yrs). We did not identify any significant between-group differences at any time period with respect to autoimmune health. For participants self-reporting changes in autoimmune health over time, ‘no obvious cause’ was the most commonly endorsed response option (~ 45%)(Additional File 5). There were no significant between-group differences in terms of factors identified as being contributors to changing autoimmune health.

**Table 7:** Autoimmune health across time periods

Type of ichthyosis	Sample	Self-reported autoimmune health at various life stages (mean±SEM§) (1 = very poor, 5 = very good)						Statistical analysis of change over time
		0-12yrs	12-18yrs	18-30yrs	30-40yrs	40-50yrs	> 50yrs	
All types combined	Only participants self-reporting for all timepoints (*n* = 85)	4.46 ± 0.08^a, b,c^	4.40 ± 0.09^d, e,f^	4.29 ± 0.09^g, h,i^	3.92 ± 0.12^a, d,g,j, k^	3.61 ± 0.14^b, e,h, j,l^	3.24 ± 0.13^c, f,I, k,l^	χ^2^[5] = 170.0, *p* < 0.001*
	Participants self-reporting for any timepoint (*n* = 93–176)	4.45 ± 0.06	4.37 ± 0.06	4.19 ± 0.07	3.90 ± 0.09	3.53 ± 0.11	3.17 ± 0.13	-
Ichthyosis vulgaris	Only participants self-reporting for all timepoints (*n* = 34)	4.53 ± 0.13^a, b^	4.44 ± 0.15^c, d^	4.32 ± 0.16^e^	4.06 ± 0.20	3.82 ± 0.20^a, c^	3.38 ± 0.20^b, d,e^	χ^2^[5] = 53.7, *p* < 0.001*
	Participants self-reporting for any timepoint (*n* = 38–63)	4.43 ± 0.11	4.27 ± 0.13	4.13 ± 0.14	4.07 ± 0.15	3.79 ± 0.17	3.24 ± 0.19	-
Autosomal recessive congenital ichthyosis (ARCI)	Only participants self-reporting for all timepoints (*n* = 20)	4.30 ± 0.21^a^	4.30 ± 0.18^b, c^	4.15 ± 0.20^d, e^	3.85 ± 0.25^f^	3.35 ± 0.32^b, d^	2.95 ± 0.28^a, c,e, f^	χ^2^[5] = 53.0, *p* < 0.001*
	Participants self-reporting for any timepoint (*n* = 21–57)	4.44 ± 0.11	4.41 ± 0.10	4.07 ± 0.12	3.68 ± 0.18	3.22 ± 0.24	2.90 ± 0.27	-
X-linked ichthyosis	Only participants self-reporting for all timepoints (*n* = 20)	4.60 ± 0.11^a^	4.55 ± 0.11^b^	4.50 ± 0.11^c^	4.05 ± 0.20	3.80 ± 0.27	3.40 ± 0.28^a, b,c^	χ^2^[5] = 40.8, *p* < 0.001*
	Participants self-reporting for any timepoint (*n* = 22–28)	4.68 ± 0.09	4.64 ± 0.09	4.61 ± 0.09	4.19 ± 0.17	3.85 ± 0.21	3.41 ± 0.25	-
Epidermolytic ichthyosis	Only participants self-reporting for all timepoints (*n* = 11)	4.27 ± 0.30	4.18 ± 0.35	4.09 ± 0.34	3.36 ± 0.36	3.09 ± 0.41	3.00 ± 0.43	χ^2^[5] = 28.4, *p* < 0.001*
	Participants self-reporting for any timepoint (*n* = 11–22)	4.48 ± 0.18	4.36 ± 0.19	4.23 ± 0.19	3.68 ± 0.24	2.94 ± 0.31	3.00 ± 0.43	-
Netherton syndrome	Only participants self-reporting for all timepoints (*n* = 0)	-	-	-	-	-	-	-
	Participants self-reporting for any timepoint (*n* = 1–7)	3.67 ± 0.61	3.83 ± 0.48	4.00 ± 0.37	3.29 ± 0.36	3.25 ± 0.25	3.00 ± 0.00	-
**Statistical analysis of between-group effects**	-	H[4] = 3.0, *p* = 0.55	H[4] = 3.7, *p* = 0.45	H[4] = 6.2, *p* = 0.18	H[4] = 8.9, *p* = 0.063	H[4] = 9.5, *p* = 0.050	H[4] = 2.4, *p* = 0.66	-

§ Standard error of the mean. Where individuals self-reported across all time periods, data were analysed by Friedman test (Bonferroni-corrected significant p-values highlighted by asterisks); significant Bonferroni-corrected pairwise comparisons by Wilcoxon Signed Ranks test are highlighted by the same superscript letters within a row. Between-group comparisons were conducted using Kruskal-Wallis H-test (Bonferroni-corrected significant p-values highlighted by asterisks); Bonferroni-corrected significant pairwise comparisons are highlighted by the same superscript Arabic numerals within a column

#### Qualitative analysis

Many participants discussed worsening joint stiffness with age, as well as symptoms of (osteo)arthritis, often starting in mid-adulthood. Some participants also described high sensitivity to allergens such as pollen, specific foods, and viruses. Individuals with ARCI self-reported that their autoimmune health was worsening with age, including a greater degree of joint pain.

### Metabolic health at different time periods

#### Quantitative analysis

Consistent with an aspect of health related to ageing, metabolic health was self-reported to become worse with later time periods for all ichthyosis subtypes (although the effect was only nominally-significant for EI) (Table 8). All mean scores on this measure were > 3/5, indicating that metabolic health was regarded as being fair-very good at all time periods for ichthyosis subtypes. Additionally, there were no significant differences between the subtypes of ichthyosis at any time period with respect to self-reported metabolic health scores. Where participants reported changing metabolic health with ageing, this was most commonly assigned to ‘no obvious cause’ (35% of participants). There were no significant between-group differences in the factors reported to be linked to changing metabolic health (Additional File 6).

**Table 8:** Metabolic health across time periods

Type of ichthyosis	Sample	Self-reported metabolic health at various life stages (mean±SEM§) (1 = very poor, 5 = very good)						Statistical analysis of change over time
		0-12yrs	12-18yrs	18-30yrs	30-40yrs	40-50yrs	> 50yrs	
All types combined	Only participants self-reporting for all timepoints (*n* = 77)	4.66 ± 0.06^a, b,c^	4.58 ± 0.07^d, e,f^	4.48 ± 0.08^g, h,i^	4.19 ± 0.11^a, d,g, j,k^	3.78 ± 0.11^b, e,h, j^	3.57 ± 0.12^c, f,I, k^	χ^2^[5] = 180.8, *p* < 0.001*
	Participants self-reporting for any timepoint (*n* = 84–159)	4.57 ± 0.05	4.49 ± 0.06	4.30 ± 0.07	4.06 ± 0.08	3.71 ± 0.09	3.52 ± 0.11	-
Ichthyosis vulgaris	Only participants self-reporting for all timepoints (*n* = 30)	4.73 ± 0.08^a, b^	4.73 ± 0.08^c, d^	4.63 ± 0.11^e, f^	4.27 ± 0.17^g^	3.90 ± 0.15^a, c,e^	3.53 ± 0.16^b, d,f, g^	χ^2^[5] = 73.9, *p* < 0.001*
	Participants self-reporting for any timepoint (*n* = 35–55)	4.53 ± 0.11	4.42 ± 0.12	4.33 ± 0.12	4.10 ± 0.15	3.84 ± 0.14	3.49 ± 0.14	-
Autosomal recessive congenital ichthyosis (ARCI)	Only participants self-reporting for all timepoints (*n* = 21)	4.62 ± 0.13^a, b^	4.48 ± 0.15^c, d^	4.29 ± 0.20^e, f^	4.05 ± 0.28	3.38 ± 0.29^a, c,e^	3.48 ± 0.26^b, d,f^	χ^2^[5] = 52.7, *p* < 0.001*
	Participants self-reporting for any timepoint (*n* = 22–54)	4.55 ± 0.11	4.47 ± 0.10	4.19 ± 0.12	4.09 ± 0.16	3.52 ± 0.20	3.45 ± 0.25	-
X-linked ichthyosis	Only participants self-reporting for all timepoints (*n* = 17)	4.53 ± 0.15^a, b^	4.47 ± 0.15	4.41 ± 0.15	4.18 ± 0.18	3.82 ± 0.20^a^	3.53 ± 0.24^b^	χ^2^[5] = 42.1, *p* < 0.001*
	Participants self-reporting for any timepoint (*n* = 18–24)	4.63 ± 0.12	4.58 ± 0.12	4.46 ± 0.13	4.08 ± 0.18	3.77 ± 0.20	3.44 ± 0.25	-
Epidermolytic ichthyosis	Only participants self-reporting for all timepoints (*n* = 9)	4.78 ± 0.15	4.56 ± 0.24	4.56 ± 0.24	4.33 ± 0.24	4.22 ± 0.22	4.00 ± 0.33	χ^2^[5] = 14.4, *p* = 0.013
	Participants self-reporting for any timepoint (*n* = 9–20)	4.68 ± 0.11	4.55 ± 0.14	4.35 ± 0.18	4.12 ± 0.19	3.80 ± 0.24	4.00 ± 0.33	-
Netherton syndrome	Only participants self-reporting for all timepoints (*n* = 0)	-	-	-	-	-	-	-
	Participants self-reporting for any timepoint (*n* = 0–6)	4.67 ± 0.21	4.83 ± 0.17	4.33 ± 0.21	3.33 ± 0.21	3.00 ± 0.00	-	-
**Statistical analysis of between-group effects**	-	H[4] = 0.32, *p* = 0.99	H[4] = 1.7, *p* = 0.79	H[4] = 2.4, *p* = 0.67	H[4] = 5.5, *p* = 0.24	H[4] = 3.8, *p* = 0.43	H[3] = 2.7, *p* = 0.45	-

§ Standard errors of the mean. Where individuals self-reported across all time periods, data were analysed by Friedman test (Bonferroni-corrected significant p-values highlighted by asterisks); significant Bonferroni-corrected pairwise comparisons by Wilcoxon Signed Ranks test are highlighted by the same superscript letters within a row. Between-group comparisons were conducted using Kruskal-Wallis H-test (Bonferroni-corrected significant p-values highlighted by asterisks); Bonferroni-corrected significant pairwise comparisons are highlighted by the same superscript Arabic numerals within a column

#### Qualitative analysis

Of the 57 participants who provided qualitative metabolic health information, > 35% identified high cholesterol as their primary concern, typically emerging in mid-adulthood and requiring management through medication combined with progressive lifestyle modifications. >20% of the respondents described either a current Type 2 diabetes diagnosis, or a family history of the condition, in their free-text responses. Additional concerns included thyroid-related issues, which commonly developed during mid-adulthood and obesity that typically fluctuated throughout life.

### Psychological/mental health at different time periods

#### Quantitative analysis

Analysis of self-reported psychological/mental health in our sample produced some interesting, and unanticipated, findings. In the overall sample, self-reported psychological/mental health remained relatively constant across the time periods assessed (Table 9). However, examining the ichthyosis subtypes individually showed distinct patterns of effects: for participants diagnosed with ARCI or EI, psychological health remained stable across time periods. In contrast, psychological health in IV and XLI varied significantly with age, with lowest scores (i.e. poorest health) reported during adolescence (12-18yrs) for IV, and in early middle-age (40-50yrs) for XLI. Mean scores between 2 and 3, indicative of poor- fair psychological health, were observed for individuals with IV or ARCI during adolescence (12-18yrs) and for Netherton syndrome from young-mid adulthood (30yrs) onwards, although in the latter case, there were a very small number of respondents. There was a single significant between-group effect, limited to adolescence, with respect to this health measure, with individuals diagnosed with either IV or ARCI self-reporting significantly worse psychological health than individuals diagnosed with XLI.

**Table 9:** Psychological/mental health across time periods

Type of ichthyosis	Sample	Self-reported psychological/mental health at various life stages (mean±SEM§) (1 = very poor, 5 = very good)						Statistical analysis of change over time
		0-12yrs	12-18yrs	18-30yrs	30-40yrs	40-50yrs	> 50yrs	
All types combined	Only participants self-reporting for all timepoints (*n* = 84)	3.67 ± 0.12	3.41 ± 0.12	3.58 ± 0.12	3.65 ± 0.12	3.58 ± 0.13	3.59 ± 0.13	χ^2^[5] = 9.5, *p* = 0.09
	Participants self-reporting for any timepoint (*n* = 88–180)	3.59 ± 0.08	3.12 ± 0.09	3.32 ± 0.09	3.51 ± 0.09	3.42 ± 0.10	3.57 ± 0.13	-
Ichthyosis vulgaris	Only participants self-reporting for all timepoints (*n* = 36)	3.61 ± 0.16	3.25 ± 0.18	3.50 ± 0.18	3.61 ± 0.18	3.72 ± 0.19	3.72 ± 0.20	χ^2^[5] = 16.3, *p* = 0.006*
	Participants self-reporting for any timepoint (*n* = 38–68)	3.52 ± 0.13	^1^2.82 ± 0.14	3.10 ± 0.14	3.56 ± 0.14	3.55 ± 0.15	3.68 ± 0.19	-
Autosomal recessive congenital ichthyosis (ARCI)	Only participants self-reporting for all timepoints (*n* = 18)	3.28 ± 0.29	3.22 ± 0.26	3.50 ± 0.29	3.78 ± 0.30	3.67 ± 0.31	3.50 ± 0.32	χ^2^[5] = 8.9, *p* = 0.11
	Participants self-reporting for any timepoint (*n* = 20–54)	3.34 ± 0.16	^2^2.92 ± 0.16	3.39 ± 0.15	3.51 ± 0.18	3.42 ± 0.21	3.40 ± 0.31	-
X-linked ichthyosis	Only participants self-reporting for all timepoints (*n* = 20)	4.10 ± 0.24	3.95 ± 0.25	3.80 ± 0.25	3.65 ± 0.23	3.25 ± 0.26	3.45 ± 0.27	χ^2^[5] = 16.9, *p* = 0.005*
	Participants self-reporting for any timepoint (*n* = 20–30)	4.03 ± 0.21	^1,2^3.87 ± 0.22	3.67 ± 0.22	3.59 ± 0.22	3.32 ± 0.24	3.45 ± 0.27	-
Epidermolytic ichthyosis	Only participants self-reporting for all timepoints (*n* = 9)	3.67 ± 0.37	3.22 ± 0.40	3.56 ± 0.34	3.78 ± 0.36	3.89 ± 0.39	3.89 ± 0.39	χ^2^[5] = 2.8, *p* = 0.73
	Participants self-reporting for any timepoint (*n* = 9–22)	3.86 ± 0.23	3.41 ± 0.24	3.27 ± 0.26	3.37 ± 0.30	3.27 ± 0.38	3.89 ± 0.39	-
Netherton syndrome	Only participants self-reporting for all timepoints (*n* = 1)	3.00 ± 0.00	3.00 ± 0.00	4.00 ± 0.00	3.00 ± 0.00	2.00 ± 0.00	2.00 ± 0.00	-
	Participants self-reporting for any timepoint (*n* = 1–6)	3.33 ± 0.42	3.50 ± 0.43	3.50 ± 0.34	3.00 ± 0.37	3.00 ± 0.41	2.00 ± 0.00	-
**Statistical analysis of between-group effects**	-	H[4] = 9.8, *p* = 0.05	H[4] = 18.3, *p* = 0.001*	H[4] = 5.2, *p* = 0.27	H[4] = 1.7, *p* = 0.78	H[4] = 1.6, *p* = 0.82	H[4] = 2.9, *p* = 0.57	-

§ Standard error of the mean. Where individuals self-reported across all time periods, data were analysed by Friedman test (Bonferroni-corrected significant p-values highlighted by asterisks); significant Bonferroni-corrected pairwise comparisons by Wilcoxon Signed Ranks test are highlighted by the same superscript letters within a row. Between-group comparisons were conducted using Kruskal-Wallis H-test (Bonferroni-corrected significant p-values highlighted by asterisks); Bonferroni-corrected significant pairwise comparisons are highlighted by the same superscript Arabic numerals within a column

Changing psychological health across the ichthyoses with age was most commonly-attributed to changes in personal circumstances (55% of participants), and there were no significant between-group effects with respect to the identification of contributory factors to changing psychological health (Additional File 7).

#### Qualitative analysis

Participants frequently described depressive symptoms beginning in adolescence as they became more aware of their skin condition. These symptoms were often lifelong, negatively impacting their ability to socialise and form relationships. Participants explained how overt childhood bullying evolved into more subtle, yet equally damaging, forms of stigmatisation in adulthood, including staring, physical avoidance and workplace discrimination. The timing and nature of these mental health challenges varied somewhat by ichthyosis subtype. Individuals with ARCI and IV experienced the most significant impact during adolescence, reporting bullying, low self-esteem, and embarrassment that substantially affected their ability to form and maintain relationships. Conversely, individuals with XLI were more likely to develop mental health issues in later life, citing negative career impacts and the emergence of comorbid conditions.

### Homeostatic health at different time periods

#### Quantitative analysis

There was a significant association between homeostatic health and age in the overall participant sample, with generally higher scores (better health) up to 30 years of age, and lower scores thereafter (Table 10). This general pattern of data was chiefly driven by a significant effect of time period in participants diagnosed with IV, and a nominally-significant effect in participants with XLI. No significant effects of time period were seen for ARCI or for EI, and such effects in Netherton syndrome could not be tested. In participants diagnosed with IV and XLI, mean scores indicated fair-very good health in this area at all time periods assessed, whereas for participants diagnosed with ARCI, EI or Netherton syndrome, mean scores indicated consistently poor-fair health in this area across time periods. Consistent with this, there were significant between-group effects across many of the time periods assessed; pairwise comparisons indicated that these effects reflected highest scores (better health) in participants with XLI, lower scores in participants with IV, and the lowest scores in participants with ARCI, EI or Netherton syndrome.

**Table 10:** Homeostatic health across time periods

Type of ichthyosis	Sample	Self-reported homeostatic health at various life stages (mean±SEM§) (1 = very poor, 5 = very good)						Statistical analysis of change over time
		0-12yrs	12-18yrs	18-30yrs	30-40yrs	40-50yrs	> 50yrs	
All types combined	Only participants self-reporting for all timepoints (*n* = 83)	3.55 ± 0.14	3.55 ± 0.14	3.57 ± 0.14^a^	3.45 ± 0.14	3.30 ± 0.14^a^	3.27 ± 0.15	χ^2^[5] = 22.0, *p* < 0.001*
	Participants self-reporting for any timepoint (*n* = 87–171)	3.32 ± 0.10	3.33 ± 0.10	3.34 ± 0.09	3.28 ± 0.10	3.21 ± 0.11	3.26 ± 0.15	-
Ichthyosis vulgaris	Only participants self-reporting for all timepoints (*n* = 35)	3.86 ± 0.18	3.83 ± 0.18	3.77 ± 0.18	3.57 ± 0.21	3.40 ± 0.21	3.43 ± 0.23	χ^2^[5] = 20.4, *p* = 0.001*
	Participants self-reporting for any timepoint (*n* = 37–63)	^4^3.49 ± 0.16	^1,5^3.50 ± 0.16	^3^3.40 ± 0.15	^4^3.33 ± 0.17	3.33 ± 0.18	3.43 ± 0.22	-
Autosomal recessive congenital ichthyosis (ARCI)	Only participants self-reporting for all timepoints (*n* = 19)	2.63 ± 0.31	2.63 ± 0.29	2.84 ± 0.32	2.84 ± 0.32	2.84 ± 0.32	2.74 ± 0.31	χ^2^[5] = 6.0, *p* = 0.31
	Participants self-reporting for any timepoint (*n* = 20–52)	^3^2.80 ± 0.17	^1,2^2.73 ± 0.16	^2^2.98 ± 0.16	^3^3.00 ± 0.18	^2^2.94 ± 0.22	2.80 ± 0.30	-
X-linked ichthyosis	Only participants self-reporting for all timepoints (*n* = 19)	4.47 ± 0.19	4.47 ± 0.18	4.47 ± 0.16	4.32 ± 0.19	4.05 ± 0.27	3.95 ± 0.31	χ^2^[5] = 13.3, *p* = 0.02
	Participants self-reporting for any timepoint (*n* = 20–29)	^1,2,3,4^4.41 ± 0.14	^2,3,4,5^4.41 ± 0.14	^1,2,3^4.34 ± 0.13	^1,2,3,4^4.23 ± 0.16	^1,2^3.92 ± 0.23	3.85 ± 0.31	-
Epidermolytic ichthyosis	Only participants self-reporting for all timepoints (*n* = 9)	2.56 ± 0.29	2.67 ± 0.29	2.56 ± 0.24	2.56 ± 0.24	2.44 ± 0.24	2.44 ± 0.24	χ^2^[5] = 2.4, *p* = 0.79
	Participants self-reporting for any timepoint (*n* = 9–21)	^2^2.74 ± 0.26	^4^2.95 ± 0.23	^1^2.81 ± 0.19	^1^2.63 ± 0.19	^1^2.38 ± 0.26	2.44 ± 0.24	-
Netherton syndrome	Only participants self-reporting for all timepoints (*n* = 1)	2.00 ± 0.00	2.00 ± 0.00	2.00 ± 0.00	2.00 ± 0.00	2.00 ± 0.00	2.00 ± 0.00	-
	Participants self-reporting for any timepoint (*n* = 1–6)	^1^2.50 ± 0.22	^3^2.83 ± 0.31	2.83 ± 0.60	^2^2.67 ± 0.49	3.00 ± 0.41	2.00 ± 0.00	-
**Statistical analysis of between-group effects**	-	H[4] = 38.0, *p* < 0.001*	H[4] = 40.4, *p* < 0.001*	H[4] = 31.7, *p* < 0.001*	H[4] = 26.1, *p* < 0.001*	H[4] = 16.5, *p* = 0.002*	H[4] = 11.1, *p* = 0.03	-

§ Standard error of the mean. Where individuals self-reported across all time periods, data were analysed by Friedman test (Bonferroni-corrected significant p-values highlighted by asterisks); significant Bonferroni-corrected pairwise comparisons by Wilcoxon Signed Ranks test are highlighted by the same superscript letters within a row. Between-group comparisons were conducted using Kruskal-Wallis H-test (Bonferroni-corrected significant p-values highlighted by asterisks); Bonferroni-corrected significant pairwise comparisons are highlighted by the same superscript Arabic numerals within a column

Where participants reported changing homeostatic health across time, the underlying contributing factors could often not be identified (~ 35% of participants)(Additional File 8). There were no between-group differences in factors identified as being contributory towards changing homeostatic health across the various time periods.

#### Qualitative analysis

Participants’ homeostatic health experiences centred primarily on difficulty maintaining body temperature, often related to an inability to sweat. Individuals with ARCI and EI often stated that temperature regulation ‘has always been a problem’, and expressed ongoing concerns about overheating, particularly during summer months. Individuals with IV reported similar experiences alongside requiring frequent hydration, while participants with XLI noted that episodes of elevated body temperature had become increasingly frequent and prolonged with age.

### Mobility at different time periods

#### Quantitative analysis

Across the overall sample, and across all subtypes, there was the expected general decrease in mobility over time periods (Table 11). Mean scores for all subtypes indicated that generally, mobility across the various time periods was regarded as fair-very good (mean scores of ≥ 3), although for both EI and Netherton syndrome, there was some evidence for impaired mobility (poor-fair) from 40 years of age onwards (mean scores of 2–3). We found evidence for significant between-group effects across time periods, consistent with a pattern of data whereby in terms of scores, XLI > IV=ARCI=Netherton syndrome > EI. Where participants reported changing mobility across time periods, the underlying contributing factors could often not be identified (~ 35% of participants)(Additional File 9). There was a significant between-group effect on the proportion of individuals reporting ‘no obvious cause’ as a contributory factor, with significantly more individuals diagnosed with XLI reporting this factor than expected (*p* < 0.05).

**Table 11:** Mobility across time periods

Type of ichthyosis	Sample	Self-reported mobility at various life stages (mean±SEM§) (1 = very poor, 5 = very good)						Statistical analysis of change over time
		0-12yrs	12-18yrs	18-30yrs	30-40yrs	40-50yrs	> 50yrs	
All types combined	Only participants self-reporting for all timepoints (*n* = 89)	4.37 ± 0.10^a, b^	4.48 ± 0.08^c, d^	4.43 ± 0.09^e, f^	4.42 ± 0.10^g^	3.76 ± 0.12^a, c,e^	3.20 ± 0.13^b, d,f, g^	χ^2^[5] = 167.1, *p* < 0.001*
	Participants self-reporting for any timepoint (*n* = 91–177)	4.26 ± 0.08	4.36 ± 0.07	4.24 ± 0.07	4.01 ± 0.08	3.67 ± 0.10	3.21 ± 0.13	-
Ichthyosis vulgaris	Only participants self-reporting for all timepoints (*n* = 38)	4.42 ± 0.15^a^	4.45 ± 0.15^b^	4.42 ± 0.15^c^	4.16 ± 0.16^d^	3.89 ± 0.16	3.42 ± 0.19^a, b,c, d^	χ^2^[5] = 57.3, *p* < 0.001*
	Participants self-reporting for any timepoint (*n* = 40–67)	^2^4.38 ± 0.13	4.36 ± 0.13	4.22 ± 0.13	^3^4.12 ± 0.13	^1^3.86 ± 0.14	3.43 ± 0.18	-
Autosomal recessive congenital ichthyosis (ARCI)	Only participants self-reporting for all timepoints (*n* = 20)	4.30 ± 0.22^a^	4.40 ± 0.18^b^	4.30 ± 0.23^c^	4.05 ± 0.28	3.85 ± 0.32	3.25 ± 0.33^a, b,c^	χ^2^[5] = 37.0, *p* < 0.001*
	Participants self-reporting for any timepoint (*n* = 20–51)	4.26 ± 0.14	4.35 ± 0.11	4.29 ± 0.13	^2^4.05 ± 0.17	^2^3.81 ± 0.24	3.25 ± 0.33	-
X-linked ichthyosis	Only participants self-reporting for all timepoints (*n* = 21)	4.71 ± 0.10^a^	4.71 ± 0.10^b^	4.62 ± 0.13^c^	4.38 ± 0.16^d^	3.86 ± 0.21	3.10 ± 0.28^a, b,c, d^	χ^2^[5] = 57.7, *p* < 0.001*
	Participants self-reporting for any timepoint (*n* = 21–31)	^1,3^4.77 ± 0.08	4.77 ± 0.08	^1^4.68 ± 0.10	^1,4^4.43 ± 0.13	^3^3.92 ± 0.20	3.10 ± 0.28	-
Epidermolytic ichthyosis	Only participants self-reporting for all timepoints (*n* = 9)	3.78 ± 0.46	4.33 ± 0.29	4.33 ± 0.17	3.67 ± 0.24	3.00 ± 0.29	2.67 ± 0.33	χ^2^[5] = 17.0, *p* = 0.004*
	Participants self-reporting for any timepoint (*n* = 9–22)	^2,3^3.45 ± 0.30	3.91 ± 0.25	^1^3.68 ± 0.24	^2,3,4^3.26 ± 0.23	^1,2,3^2.63 ± 0.27	2.67 ± 0.33	-
Netherton syndrome	Only participants self-reporting for all timepoints (*n* = 1)	2.00 ± 0.00	4.00 ± 0.00	4.00 ± 0.00	3.00 ± 0.00	2.00 ± 0.00	1.00 ± 0.00	-
	Participants self-reporting for any timepoint (*n* = 1–6)	^1^3.33 ± 0.49	4.00 ± 0.45	3.83 ± 0.40	^1^3.00 ± 0.37	2.75 ± 0.48	1.00 ± 0.00	-
**Statistical analysis of between-group effects**	-	H[4] = 22.3, *p* < 0.001*	H[4] = 12.2, *p* = 0.013	H[4] = 16.4, *p* = 0.003*	H[4] = 22.0, *p* < 0.001*	H[4] = 18.7, *p* < 0.001*	H[4] = 5.6, *p* = 0.23	-

§ Standard error of the mean. Where individuals self-reported across all time periods, data were analysed by Friedman test (Bonferroni-corrected significant p-values highlighted by asterisks); significant Bonferroni-corrected pairwise comparisons by Wilcoxon Signed Ranks test are highlighted by the same superscript letters within a row. Between-group comparisons were conducted using Kruskal-Wallis H-test (Bonferroni-corrected significant p-values highlighted by asterisks); Bonferroni-corrected significant pairwise comparisons are highlighted by the same superscript Arabic numerals within a column

#### Qualitative analysis

Participants consistently reported mobility restrictions caused by tight, or dry, skin, that typically worsened at later time periods. These mobility issues were compounded by joint stiffness, pain, and arthritis, which commonly began around 35 years of age. Many individuals emphasized the importance of self-management strategies, including maintaining physical activity when possible, and regularly applying topical treatments to preserve mobility. Individuals with EI reported particularly severe mobility limitations, with even short-distance walking proving difficult, and prolonged walking causing skin blisters. In contrast, few participants with XLI experienced such extreme limitations, typically attributing their mobility decline to normal ageing or moderate skin cracking.

### Social relationships at different time periods

#### Quantitative analysis

Across the overall sample, there was a significant change in the quality of social relationships with time period, with poorer relationships reported during childhood (< 18yrs), and an improvement from early adulthood onwards, although relationship quality did seem to drop again in the oldest age group assessed (Table 12). This pattern of data was somewhat similar across IV, ARCI and EI subtypes; however, the finding in ARCI was only nominally-significant, and for EI, social relationships at the earliest time period assessed (0-12yrs) were regarded as relatively positive. For participants diagnosed with XLI, the quality of social relationships across time periods tended to be relatively stable. Social relationships were regarded as being fair-good (mean scores of 3–4) for most subtypes and time periods, although lower mean scores (≤ 3), indicative of poor-fair relationships, were reported for EI during adolescence (12-18yrs) and for Netherton syndrome across childhood and adolescence (< 18yrs).

**Table 12:** Social relationships across time periods

Type of ichthyosis	Sample	Self-reported social relationships at various life stages (mean±SEM§) (1 = very poor, 5 = very good)						Statistical analysis of change over time
		0-12yrs	12-18yrs	18-30yrs	30-40yrs	40-50yrs	> 50yrs	
All types combined	Only participants self-reporting for all timepoints (*n* = 87)	3.30 ± 0.13^a, b^	3.24 ± 0.12^c, d,e^	3.63 ± 0.11^c^	3.85 ± 0.11^a, d^	3.77 ± 0.11^b, e^	3.62 ± 0.12	χ^2^[5] = 44.9, *p* < 0.001*
	Participants self-reporting for any timepoint (*n* = 89–175)	3.32 ± 0.09	3.16 ± 0.09	3.59 ± 0.08	3.75 ± 0.09	3.75 ± 0.09	3.63 ± 0.12	-
Ichthyosis vulgaris	Only participants self-reporting for all timepoints (*n* = 39)	3.26 ± 0.17	3.15 ± 0.18^a, b,c^	3.59 ± 0.16^a^	3.85 ± 0.16^b^	3.79 ± 0.16^c^	3.56 ± 0.18	χ^2^[5] = 25.3, *p* < 0.001*
	Participants self-reporting for any timepoint (*n* = 40–67)	3.26 ± 0.14	2.97 ± 0.14	3.35 ± 0.14	3.72 ± 0.14	3.76 ± 0.15	3.58 ± 0.17	-
Autosomal recessive congenital ichthyosis (ARCI)	Only participants self-reporting for all timepoints (*n* = 19)	2.89 ± 0.30	3.21 ± 0.25	3.68 ± 0.28	3.68 ± 0.29	3.63 ± 0.30	3.58 ± 0.30	χ^2^[5] = 12.8, *p* = 0.026
	Participants self-reporting for any timepoint (*n* = 20–50)	3.31 ± 0.18	3.37 ± 0.16	3.94 ± 0.14	3.87 ± 0.17	3.74 ± 0.20	3.60 ± 0.28	-
X-linked ichthyosis	Only participants self-reporting for all timepoints (*n* = 19)	3.74 ± 0.30	3.74 ± 0.24	3.79 ± 0.24	3.89 ± 0.24	3.79 ± 0.26	3.58 ± 0.29	χ^2^[5] = 3.7, *p* = 0.60
	Participants self-reporting for any timepoint (*n* = 19–31)	3.52 ± 0.24	3.52 ± 0.21	3.73 ± 0.19	3.71 ± 0.19	3.81 ± 0.21	3.58 ± 0.29	-
Epidermolytic ichthyosis	Only participants self-reporting for all timepoints (*n* = 9)	3.56 ± 0.41	2.67 ± 0.37	3.33 ± 0.29	4.11 ± 0.20	3.89 ± 0.26	4.00 ± 0.29	χ^2^[5] = 16.4, *p* = 0.006*
	Participants self-reporting for any timepoint (*n* = 9–22)	3.41 ± 0.25	2.91 ± 0.26	3.32 ± 0.23	3.63 ± 0.24	3.50 ± 0.27	4.00 ± 0.29	-
Netherton syndrome	Only participants self-reporting for all timepoints (*n* = 1)	2.00 ± 0.00	3.00 ± 0.00	4.00 ± 0.00	4.00 ± 0.00	4.00 ± 0.00	4.00 ± 0.00	-
	Participants self-reporting for any timepoint (*n* = 1–6)	2.67 ± 0.33	2.67 ± 0.33	3.67 ± 0.21	4.00 ± 0.26	4.25 ± 0.25	4.00 ± 0.00	-
**Statistical analysis of between-group effects**	-	H[4] = 3.8, *p* = 0.43	H[4] = 8.4, *p* = 0.08	H[4] = 11.2, *p* = 0.03	H[4] = 1.2, *p* = 0.87	H[4] = 2.1, *p* = 0.72	H[4] = 0.9, *p* = 0.92	-

§ Standard error of the mean. Where individuals self-reported across all time periods, data were analysed by Friedman test (Bonferroni-corrected significant p-values highlighted by asterisks); significant Bonferroni-corrected pairwise comparisons by Wilcoxon Signed Ranks test are highlighted by the same superscript letters within a row. Between-group comparisons were conducted using Kruskal-Wallis H-test (Bonferroni-corrected significant p-valuses highlighted by asterisks); Bonferroni-corrected significant pairwise comparisons are highlighted by the same superscript Arabic numerals within a column

The main contributory factor identified as being responsible for changing social relationships over time periods across the overall sample was ‘changing personal circumstances’ (~ 45% of participants)(Additional File 10). There were no significant between-group differences in factors contributing to changing social relationships.

#### Qualitative analysis

Participants frequently described experiences of bullying and feeling ‘othered’ during childhood and adolescence, which hindered their ability to form friendships. These early social difficulties often persisted into early adulthood, creating challenges in developing romantic relationships and limiting social interactions more broadly. As participants aged, however, many reported establishing strong support networks, particularly by mid-adulthood, which increased their resilience to public stigmatisation. Despite this improved social support, participants consistently described a cyclical relationship between skin issues, embarrassment, confidence, and social engagement, with worse skin conditions leading to greater embarrassment, reduced confidence, and consequently less socialising. Participants with IV specifically described intense social isolation during childhood, though many found social engagement became easier as their skin improved through effective treatment in early-to-mid adulthood.

### Future concerns about health

#### Quantitative analysis

Across the overall sample, participants were most concerned about worsening of their skin condition and other medical conditions over time (mean scores > 3.5/5). Individuals diagnosed with ARCI and XLI were relatively less concerned with worsening of any extracutaneous conditions or their skin condition respectively (Table 13). Individuals with Netherton syndrome were also highly concerned about future aid with skincare, and about future healthcare provision. There were no significant differences between ichthyosis subtypes in terms of future concerns.

**Table 13:** Future concerns about health

Type of ichthyosis	Future concerns about health (mean±SEM§) (1 = not at all concerned, 5 = extremely concerned)				
	Worsening skin condition	Worsening of other medical or psychological conditions	Inability of relatives/carers to help with skincare	Access to sufficient healthcare provision	Menopause and reproductive health
All types combined (*n* = 162)	3.58 ± 0.11	3.64 ± 0.11	3.16 ± 0.12	3.34 ± 0.11	3.03 ± 0.14
Ichthyosis vulgaris (*n* = 62)	3.76 ± 0.15	3.67 ± 0.16	3.34 ± 0.21	3.48 ± 0.18	3.10 ± 0.25
Autosomal recessive congenital ichthyosis (ARCI)(*n* = 47)	3.51 ± 0.21	3.48 ± 0.22	2.89 ± 0.23	3.26 ± 0.20	2.95 ± 0.23
X-linked ichthyosis (*n* = 28)	2.96 ± 0.25	3.50 ± 0.29	2.83 ± 0.32	2.96 ± 0.29	2.92 ± 0.50
Epidermolytic ichthyosis (*n* = 20)	3.85 ± 0.31	3.85 ± 0.26	3.45 ± 0.30	3.37 ± 0.26	3.10 ± 0.36
Netherton syndrome (*n* = 5)	4.40 ± 0.40	4.80 ± 0.20	3.80 ± 0.80	4.40 ± 0.60	3.00 ± 0.84
**Statistical analysis of between-group effects**	H[4] = 10.0, *p* = 0.04	H[4] = 4.9, *p* = 0.30	H[4] = 4.7, *p* = 0.32	H[4] = 5.9, *p* = 0.21	H[4] = 0.4, *p* = 0.98

§ Standard error of the mean. Between-group comparisons were conducted using Kruskal-Wallis H-test (Bonferroni-corrected significant p-values highlighted by asterisks); Bonferroni-corrected significant pairwise comparisons are highlighted by the same superscript Arabic numerals within a column

#### Qualitative analysis

Participants’ concerns around the worsening of skin with age typically related to a lack of readily available clinical information (and therefore patient information) about how the ichthyoses manifest throughout life. This was also closely related to the limited knowledge of healthcare professionals; participants expressed their frustration with a reliance on the patient to provide up-to-date information about their condition, and little long-term planning and support from clinicians. Many future concerns reflected sex-specific experiences of ichthyosis. Female participants openly discussed apprehensions about the effects of perimenopause, including potential impacts of hormone replacement therapy (HRT). Younger participants voiced uncertainty about family planning decisions and how their condition might affect pregnancy outcomes. The most frequently discussed priorities for future research were with respect to improving current treatments (and enhancing access to improved treatments), identifying and implementing support needs for psychological health and wellbeing, and investigating the prevalence and effects of comorbid conditions (and strategies for monitoring of these). Importantly, the effects of perimenopause were also highlighted as a research priority by female participants.

## Discussion

Previous research on the ichthyoses has focused on assessing (extra)cutaneous symptoms within specific subtypes at specific developmental time periods and comparing these to those exhibited by unaffected individuals. In this novel analysis, we obtained self-reported data on an array of medical conditions for multiple subtypes of congenital ichthyosis across developmental time periods and directly compared between subtypes and time periods. Our approach allows quantitative analysis of individuals’ perceptions of their condition(s), and the addition of qualitative data allows for a richer description of patient feelings/concerns.

We managed to recruit a large participant panel from across the world, with the number of respondents per ichthyosis subtype approximating to the relative prevalence of the various conditions within the general population [3]. The sample was female-biased, consistent with other online studies investigating health phenotypes [18]. This bias probably reflects a combination of increased social media use and engagement with medical professionals in females, as well as condition-specific factors. However, a reasonable number of males with ichthyosis did respond, notably for XLI, which almost exclusively affects this sex. The sample was also largely USA and UK-based, and of White ethnicity. Although attempts were made to advertise the survey worldwide, these biases probably reflect the membership demographics of existing ichthyosis charities and large patient support groups. As such, the present findings should be interpreted cautiously with respect to their generalisability to residents from non-European/USA countries with differing healthcare systems and cultures, and to all other non-White ethnic groups.

Our first main finding was that all subtypes of ichthyosis can have significant, lifelong, effects on quality of life, but that these effects tend to diminish over time. This lessening impact may be due to multiple interacting factors including: (i) the availability of, or access to, better treatments, (ii) increased knowledge and social support (e.g. patient forums and charities), and (iii) optimized coping strategies [19, 20]. Unsurprisingly, more severe subtypes of ichthyosis (ARCI, EI and Netherton syndrome) were associated with more extensive impacts on quality of life, particularly in childhood. This finding reinforces the need for medical and social support for individuals with these subtypes as early as possible. The pattern of ‘quality of life’ data was mirrored by the pattern of ‘skin severity’ data, consistent with the intuitive notion that subtypes of ichthyosis with a more severe skin phenotype impact to a greater extent upon quality of life [21]. Interestingly, in individuals diagnosed with Netherton syndrome, changes in skin health appeared particularly responsive to changes in medication/treatments.

Participants diagnosed with ARCI, EI or Netherton syndrome reported ongoing problems with homeostatic health (e.g. temperature regulation) throughout life, and participants with these conditions also tended to exhibit problems with autoimmune health from middle age onwards (40- 50yrs). In addition, the oldest individuals diagnosed with ARCI tended to report problems with bone health, and there was also, less robust, evidence for an age-related effect on bone health in Netherton syndrome. In comparison, individuals diagnosed with XLI may be relatively protected against bone- related problems in later life as a consequence of being male and therefore not experiencing the loss of osteoprotective oestrogen post-menopausally [22]. Mobility issues appeared particularly significant for older individuals diagnosed with EI, and to a lesser extent were identified by older individuals affected by IV, ARCI or Netherton syndrome. Together, these findings suggest that clinical advice regarding homeostatic maintenance (e.g. body temperature regulation, fluid balance) should be provided early in life for individuals diagnosed with more severe ichthyosis subtypes, and that individuals diagnosed with such subtypes should be monitored from middle-age onwards for bone health, autoimmune conditions, and mobility issues, and advice and/or interventions provided where necessary. Interestingly, participants did not tend to report significant health concerns with respect to cardiovascular or metabolic function across time periods, suggesting that these areas should not be a priority for screening. However, it is possible that, whilst most individuals with ichthyosis experience no, or minor, adverse effects with respect to these aspects of health, small numbers of individuals might experience significant adverse effects or such adverse effects may be asymptomatic (and hence remain unreported). For example, a robust association has been identified between XLI and cardiac arrhythmias which can have significant downstream effects on general health and which need to be identified early via screening [11].

As expected, psychological health was self-reported as being consistently lower, and relatively stable, across the time periods assessed for the more severe forms of ichthyosis. In the milder forms of ichthyosis, psychological health tended to fluctuate across time periods, with most adverse effects reported for IV during adolescence and for XLI during middle-age. These results imply that targeted psychological, pharmacological, or social therapeutic interventions, addressing personal circumstances, administered during early-life/adolescence for most ichthyoses, and also during early middle-age in XLI, may be clinically-efficacious. Social relationships were identified as being particularly problematic for individuals with EI during adolescence and for individuals with Netherton syndrome throughout childhood; psychological and family therapies offered early for these ichthyosis subtypes may provide clinical, educational and social benefit.

In terms of future concerns, individuals with all ichthyosis subtypes were worried about worsening of their skin condition, and worsening of other, associated, medical conditions. To alleviate these concerns, clinicians, scientists and support groups should aim to ensure that individuals diagnosed with ichthyosis are: routinely provided access to the best-available treatments, regularly updated about emerging therapeutic options, and kept informed about relevant clinical trials and the outcomes of such trials.

Although the present study is novel and has numerous strengths, it is limited in various ways: (i) self-reported diagnoses lack objective clinical verification and participants may have mis-interpreted medical terminology (although individuals diagnosed with rare diseases tend to be well-informed about their condition [23], and exemplars related to aspects of health were provided in the survey), (ii) while analysis at various time periods is undoubtedly desirable in the current absence of prospective cohort studies, retrospective reporting of health-related measures may introduce memory-related biases and errors, which may be more prominent in individuals with conditions affecting cognitive or mental health, (iii) although we have attempted to identify factors involved in health changes over time, the mechanisms underlying these are uncertain and remain to be clarified, and (iv) there may be various response biases e.g. whereby individuals with more severe symptoms are motivated to participate; however, presumably these biases would pertain across ichthyosis subtypes meaning that between- group inferences may still be reliably drawn.

## Conclusions

In summary, we have obtained a novel, large, dataset on a multitude of health measures across a number of subtypes of ichthyoses and across multiple time periods. These data highlight the most significant health problems affecting individuals with ichthyosis, and indicate the conditions and time periods where clinical intervention should be prioritised. Conventional longitudinal analyses in individuals with epidermal differentiation disorders should be undertaken to confirm and extend the current findings.

## Electronic Supplementary Material

Below is the link to the electronic supplementary material.

Supplementary Material 1: Additional File 1. Quantitative data from online survey

Supplementary Material 2: Additional File 2. Content analysis map for qualitative data

Supplementary Material 3: Additional File 3. Factors contributing to changes in bone health across time periods

Supplementary Material 4: Additional File 4. Factors contributing to changes in cardiovascular health across time periods

Supplementary Material 5: Additional File 5. Factors contributing to changes in autoimmune health across time periods

Supplementary Material 6: Additional File 6. Factors contributing to changes in metabolic health across time periods

Supplementary Material 7: Additional File 7. Factors contributing to changes in psychological/mental health across time periods

Supplementary Material 8: Additional File 8. Factors contributing to changes in homeostatic health across time periods

Supplementary Material 9: Additional File 9. Factors contributing to changes in mobility across time periods

Supplementary Material 10: Additional File 10. Factors contributing to changes in social relationships across time periods

## Data Availability

All data generated or analysed during this study are included in this published article and its supplementary information files.

## Abbreviations

ARCI: Autosomal recessive congenital ichthyosis
EI: Epidermolytic ichthyosis
FLG: Filaggrin
IV: Ichthyosis vulgaris
SEM: Standard error of the mean
STS: Steroid sulfatase
UK: United Kingdom
URL: Uniform Resource Locator
USA: United States of America
XLI: X-linked ichthyosis
