# Supplementary material for: A mixed-methods online survey approach using retrospective self-reporting to characterise congenital ichthyoses across age groups
Source: Orphanet J Rare Dis. 2026 Apr 18;21:209. doi: 10.1186/s13023-026-04358-7 (PMC13224449; doi:10.1186/s13023-026-04358-7)
Supplement: Supplementary file 2 — Supplementary Material 2: Additional File 2. Content analysis map for qualitative data [file 13023_2026_4358_MOESM2_ESM.docx]

**Additional File 2.** Content Analysis Map

|  | **Factor 1** | **Factor 2** | **Factor 3** |
| --- | --- | --- | --- |
| **Impact of Ichthyosis** | Stigma/bullying | Skin changing with age | Self esteem |
| **Skin Condition** | Skin changing with age | Climate | Positive effects of treatment |
| **Bone Health** | Bone pain | Arthritis | Worsening bone density |
| **Cardiovascular Health** | Hypertension | Managed with medication or surgery | Arrhythmia |
| **Autoimmune Health** | Arthritis | Other medical conditions | Sore joints/muscles |
| **Metabolic Health** | High cholesterol | Diabetic/pre-diabetic | Self-management/lifestyle changes |
| **Psychological Health** | Psychological health/wellbeing | Stigma/bullying | Childhood mental health |
| **Homeostatic Health** | Needing to maintain body temperature | Overheating | Inability to sweat |
| **Mobility** | Reduced flexibility/mobility | Sore joints/muscles | Arthritis |
| **Social Interactions** | Loneliness/limited social interactions | Forming relationships | Support from friends/family |
| **Further Comments** | Psychological health/wellbeing | Living with/managing condition | Stigma/bullying |
| **Healthcare Provisions** | Treatment availability | Limited access to HCP support | More HCP training/awareness |
| **Future Concerns** | Skin changing with age | Ability to care for skin in old age | Menopause |
| **Future Research** | Improvements to treatment | Psychological health/wellbeing | Comorbid conditions |
