# Supplementary material for: A mixed-methods online survey approach using retrospective self-reporting to characterise congenital ichthyoses across age groups
Source: Orphanet J Rare Dis. 2026 Apr 18;21:209. doi: 10.1186/s13023-026-04358-7 (PMC13224449; doi:10.1186/s13023-026-04358-7)
Supplement: Supplementary file 3 — Supplementary Material 3: Additional File 3. Factors contributing to changes in bone health across time periods [file 13023_2026_4358_MOESM3_ESM.docx]

**Additional File 3.** Factors contributing to changes in bone health across time periods

| **Type of ichthyosis** | **Number of participants reporting changing bone condition** | **Number (%) of participants reporting factor as contributory towards changing bone condition^[[1]](#footnote-1)^§** | | | | | | |
| --- | --- | --- | --- | --- | --- | --- | --- | --- |
|  |  | **Change in self-care** | **Change in personal circumstances** | **Change in living conditions** | **Change in medication or treatments** | **No obvious cause** | **Changes in medical or scientific advice** | **Other** |
| All types combined | 124 | 5 (4.0%) | 13 (10.5%) | 4 (3.2%) | 23 (18.5%) | 42 (33.9%) | 6 (4.8%) | 55 (44.4%) |
| Ichthyosis vulgaris | 39 | 0 (0.0%) | 5 (12.8%) | 0 (0.0%) | 3 (7.7%) | 13 (33.3%) | 3 (7.7%) | 21 (53.8%) |
| Autosomal Recessive Congenital Ichthyosis (ARCI) | 40 | 2 (5.0%) | 3 (7.5%) | 2 (5.0%) | 9 (22.5%) | 12 (30.0%) | 2 (5.0%) | 17 (42.5%) |
| X-linked ichthyosis | 19 | 0 (0.0%) | 2 (10.5%) | 0 (0.0%) | 2 (10.5%) | 6 (31.6%) | 0 (0.0%) | 9 (47.4%) |
| Epidermolytic ichthyosis | 20 | 2 (10.0%) | 2 (10.0%) | 1 (5.0%) | 7 (35.0%) | 8 (40.0%) | 1 (5.0%) | 7 (35.0%) |
| Netherton syndrome | 6 | 1 (16.7%) | 1 (16.7%) | 1 (16.7%) | 2 (33.3%) | 3 (50.0%) | 0 (0.0%) | 1 (16.7%) |
| **Statistical analysis of between-group effects** | - | χ^2^[4]=6.9, p=0.14 | χ^2^[4]=0.86, p=0.93 | χ^2^[4]=6.0, p=0.20 | χ^2^[4]=8.7, p=0.07 | χ^2^[4]=1.4, p=0.10 | χ^2^[4]=2.0, p=0.74 | χ^2^[4]=4.1, p=0.39 |

1. § Between-group effects analysed using chi-squared test, with significant Bonferroni-corrected p-values indicated by asterisks. [↑](#footnote-ref-1)
