# Supplementary material for: A mixed-methods online survey approach using retrospective self-reporting to characterise congenital ichthyoses across age groups
Source: Orphanet J Rare Dis. 2026 Apr 18;21:209. doi: 10.1186/s13023-026-04358-7 (PMC13224449; doi:10.1186/s13023-026-04358-7)
Supplement: Supplementary file 4 — Supplementary Material 4: Additional File 4. Factors contributing to changes in cardiovascular health across time periods [file 13023_2026_4358_MOESM4_ESM.docx]

**Additional File 4.** Factors contributing to changes in cardiovascular health across time periods

| **Type of ichthyosis** | **Number of participants reporting changing cardiovascular condition** | **Number (%) of participants reporting factor as contributory towards changing cardiovascular condition^[[1]](#footnote-1)^§** | | | | | | |
| --- | --- | --- | --- | --- | --- | --- | --- | --- |
|  |  | **Change in self-care** | **Change in personal circumstances** | **Change in living conditions** | **Change in medication or treatments** | **No obvious cause** | **Changes in medical or scientific advice** | **Other** |
| All types combined | 122 | 10 (8.2%) | 25 (28.7%) | 8 (6.6%) | 18 (14.8%) | 40 (32.8%) | 9 (7.4%) | 33 (27.0%) |
| Ichthyosis vulgaris | 44 | 3 (6.8%) | 9 (20.5%) | 3 (6.8%) | 5 (11.4%) | 10 (22.7%) | 5 (11.4%) | 16 (36.4%) |
| Autosomal Recessive Congenital Ichthyosis (ARCI) | 35 | 5 (14.3%) | 12 (34.3%) | 4 (11.4%) | 6 (17.1%) | 12 (34.3%) | 2 (5.7%) | 8 (22.9%) |
| X-linked ichthyosis | 23 | 1 (4.3%) | 8 (34.8%) | 1 (4.3%) | 2 (8.7%) | 8 (34.8%) | 1 (4.3%) | 6 (26.1%) |
| Epidermolytic ichthyosis | 14 | 1 (7.1%) | 5 (35.7%) | 0 (0.0%) | 4 (28.6%) | 6 (42.9%) | 1 (7.1%) | 2 (14.3%) |
| Netherton syndrome | 6 | 0 (0.0%) | 1 (16.7%) | 0 (0.0%) | 1 (16.7%) | 4 (66.7%) | 0 (0.0%) | 1 (16.7%) |
| **Statistical analysis of between-group effects** | - | χ^2^[4]=2.8, p=0.58 | χ^2^[4]=3.2, p=0.53 | χ^2^[4]=3.0, p=0.57 | χ^2^[4]=3.4, p=0.50 | χ^2^[4]=5.9, p=0.21 | χ^2^[4]=2.0, p=0.75 | χ^2^[4]=3.7, p=0.44 |

1. § Between-group effects analysed using chi-squared test, with significant Bonferroni-corrected p-values indicated by asterisks. [↑](#footnote-ref-1)
