# Supplementary material for: A mixed-methods online survey approach using retrospective self-reporting to characterise congenital ichthyoses across age groups
Source: Orphanet J Rare Dis. 2026 Apr 18;21:209. doi: 10.1186/s13023-026-04358-7 (PMC13224449; doi:10.1186/s13023-026-04358-7)
Supplement: Supplementary file 5 — Supplementary Material 5: Additional File 5. Factors contributing to changes in autoimmune health across time periods [file 13023_2026_4358_MOESM5_ESM.docx]

**Additional File 5.** Factors contributing to changes in autoimmune health across time periods

| **Type of ichthyosis** | **Number of participants reporting changing autoimmune condition** | **Number (%) of participants reporting factor as contributory towards changing autoimmune condition^[[1]](#footnote-1)^§** | | | | | | |
| --- | --- | --- | --- | --- | --- | --- | --- | --- |
|  |  | **Change in self-care** | **Change in personal circumstances** | **Change in living conditions** | **Change in medication or treatments** | **No obvious cause** | **Changes in medical or scientific advice** | **Other** |
| All types combined | 125 | 10 (8.0%) | 22 (17.6%) | 6 (4.8%) | 13 (10.4%) | 54 (43.2%) | 6 (4.8%) | 44 (35.2%) |
| Ichthyosis vulgaris | 47 | 3 (6.4%) | 5 (10.6%) | 1 (2.1%) | 3 (6.4%) | 20 (42.6%) | 2 (4.3%) | 20 (42.6%) |
| Autosomal Recessive Congenital Ichthyosis (ARCI) | 38 | 6 (15.8%) | 10 (26.3%) | 4 (10.5%) | 6 (15.8%) | 14 (36.8%) | 2 (5.3%) | 13 (34.2%) |
| X-linked ichthyosis | 19 | 0 (0.0%) | 3 (15.8%) | 1 (5.3%) | 1 (5.3%) | 7 (36.8%) | 0 (0.0%) | 8 (42.1%) |
| Epidermolytic ichthyosis | 15 | 1 (6.7%) | 4 (26.7%) | 0 (0.0%) | 1 (6.7%) | 8 (53.3%) | 2 (13.3%) | 3 (20.0%) |
| Netherton syndrome | 6 | 0 (0.0%) | 0 (0.0%) | 0 (0.0%) | 2 (33.3%) | 5 (83.3%) | 0 (0.0%) | 0 (0.0%) |
| **Statistical analysis of between-group effects** | - | χ^2^[4]=5.5, p=0.24 | χ^2^[4]=5.7, p=0.22 | χ^2^[4]=4.5, p=0.34 | χ^2^[4]=6.2, p=0.19 | χ^2^[4]=5.5, p=0.24 | χ^2^[4]=3.7, p=0.45 | χ^2^[4]=6.3, p=0.18 |

1. § Between-group effects analysed using chi-squared test, with significant Bonferroni-corrected p-values indicated by asterisks. [↑](#footnote-ref-1)
