# Supplementary material for: A mixed-methods online survey approach using retrospective self-reporting to characterise congenital ichthyoses across age groups
Source: Orphanet J Rare Dis. 2026 Apr 18;21:209. doi: 10.1186/s13023-026-04358-7 (PMC13224449; doi:10.1186/s13023-026-04358-7)
Supplement: Supplementary file 6 — Supplementary Material 6: Additional File 6. Factors contributing to changes in metabolic health across time periods [file 13023_2026_4358_MOESM6_ESM.docx]

**Additional File 6.** Factors contributing to changes in metabolic health across time periods

| **Type of ichthyosis** | **Number of participants reporting changing metabolic condition** | **Number (%) of participants reporting factor as contributory towards changing metabolic condition^[[1]](#footnote-1)^§** | | | | | | |
| --- | --- | --- | --- | --- | --- | --- | --- | --- |
|  |  | **Change in self-care** | **Change in personal circumstances** | **Change in living conditions** | **Change in medication or treatments** | **No obvious cause** | **Changes in medical or scientific advice** | **Other** |
| All types combined | 116 | 15 (12.9%) | 25 (21.6%) | 5 (4.3%) | 23 (19.8%) | 39 (33.6%) | 10 (8.6%) | 34 (29.3%) |
| Ichthyosis vulgaris | 43 | 7 (16.3%) | 7 (16.3%) | 0 (0.0%) | 7 (16.3%) | 8 (18.6%) | 7 (16.3%) | 17 (39.5%) |
| Autosomal Recessive Congenital Ichthyosis (ARCI) | 34 | 4 (11.8%) | 12 (35.3%) | 3 (8.8%) | 8 (23.5%) | 13 (38.2%) | 3 (8.8%) | 8 (23.5%) |
| X-linked ichthyosis | 20 | 1 (5.0%) | 3 (15.0%) | 1 (5.0%) | 2 (10.0%) | 8 (40.0%) | 0 (0.0%) | 7 (35.0%) |
| Epidermolytic ichthyosis | 13 | 2 (15.4%) | 2 (15.4%) | 1 (7.7%) | 5 (38.5%) | 6 (46.2%) | 0 (0.0%) | 1 (7.7%) |
| Netherton syndrome | 6 | 1 (16.7%) | 1 (16.7%) | 0 (0.0%) | 1 (16.7%) | 4 (66.7%) | 0 (0.0%) | 1 (16.7%) |
| **Statistical analysis of between-group effects** | - | χ^2^[4]=1.7, p=0.79 | χ^2^[4]=5.4, p=0.25 | χ^2^[4]=4.3, p=0.37 | χ^2^[4]=4.7, p=0.32 | χ^2^[4]=8.9, p=0.06 | χ^2^[4]=6.9, p=0.14 | χ^2^[4]=6.4, p=0.17 |

1. § Between-group effects analysed using chi-squared test, with significant Bonferroni-corrected p-values indicated by asterisks. [↑](#footnote-ref-1)
