# Supplementary material for: A mixed-methods online survey approach using retrospective self-reporting to characterise congenital ichthyoses across age groups
Source: Orphanet J Rare Dis. 2026 Apr 18;21:209. doi: 10.1186/s13023-026-04358-7 (PMC13224449; doi:10.1186/s13023-026-04358-7)
Supplement: Supplementary file 7 — Supplementary Material 7: Additional File 7. Factors contributing to changes in psychological/mental health across time periods [file 13023_2026_4358_MOESM7_ESM.docx]

**Additional File 7.** Factors contributing to changes in psychological/mental health across time periods

| **Type of ichthyosis** | **Number of participants reporting changing psychological condition** | **Number (%) of participants reporting factor as contributory towards changing psychological condition^[[1]](#footnote-1)^§** | | | | | | |
| --- | --- | --- | --- | --- | --- | --- | --- | --- |
|  |  | **Change in self-care** | **Change in personal circumstances** | **Change in living conditions** | **Change in medication or treatments** | **No obvious cause** | **Changes in medical or scientific advice** | **Other** |
| All types combined | 146 | 42 (28.8%) | 82 (56.2%) | 30 (20.5%) | 39 (26.7%) | 24 (16.4%) | 18 (12.3%) | 33 (22.6%) |
| Ichthyosis vulgaris | 57 | 15 (26.3%) | 32 (56.1%) | 14 (24.6%) | 14 (24.6%) | 8 (14.0%) | 8 (14.0%) | 13 (22.8%) |
| Autosomal Recessive Congenital Ichthyosis (ARCI) | 43 | 18 (41.9%) | 27 (62.8%) | 9 (20.9%) | 14 (32.6%) | 5 (11.6%) | 6 (14.0%) | 10 (23.3%) |
| X-linked ichthyosis | 23 | 2 (8.7%) | 10 (43.5%) | 3 (13.0%) | 5 (21.7%) | 7 (30.4%) | 1 (4.3%) | 4 (17.4%) |
| Epidermolytic ichthyosis | 18 | 6 (33.3%) | 10 (55.6%) | 3 (16.7%) | 6 (33.3%) | 4 (22.2%) | 3 (16.7%) | 4 (22.2%) |
| Netherton syndrome | 5 | 1 (20.0%) | 3 (60.0%) | 1 (20.0%) | 0 (0.0%) | 0 (0.0%) | 0 (0.0%) | 2 (40.0%) |
| **Statistical analysis of between-group effects** | - | χ^2^[4]=8.7, p=0.07 | χ^2^[4]=2.3, p=0.68 | χ^2^[4]=1.5, p=0.82 | χ^2^[4]=3.4, p=0.49 | χ^2^[4]=5.7, p=0.23 | χ^2^[4]=2.6, p=0.62 | χ^2^[4]=1.2, p=0.87 |

1. § Between-group effects analysed using chi-squared test, with significant Bonferroni-corrected p-values indicated by asterisks. [↑](#footnote-ref-1)
