# Supplementary material for: A mixed-methods online survey approach using retrospective self-reporting to characterise congenital ichthyoses across age groups
Source: Orphanet J Rare Dis. 2026 Apr 18;21:209. doi: 10.1186/s13023-026-04358-7 (PMC13224449; doi:10.1186/s13023-026-04358-7)
Supplement: Supplementary file 8 — Supplementary Material 8: Additional File 8. Factors contributing to changes in homeostatic health across time periods [file 13023_2026_4358_MOESM8_ESM.docx]

**Additional File 8.** Factors contributing to changes in homeostatic health across time periods

| **Type of ichthyosis** | **Number of participants reporting changing homeostatic condition** | **Number (%) of participants reporting factor as contributory towards changing homeostatic condition^[[1]](#footnote-1)^§** | | | | | | |
| --- | --- | --- | --- | --- | --- | --- | --- | --- |
|  |  | **Change in self-care** | **Change in personal circumstances** | **Change in living conditions** | **Change in medication or treatments** | **No obvious cause** | **Changes in medical or scientific advice** | **Other** |
| All types combined | 118 | 29 (24.6%) | 17 (14.4%) | 21 (17.8%) | 15 (12.7%) | 41 (34.7%) | 9 (7.6%) | 30 (25.4%) |
| Ichthyosis vulgaris | 43 | 12 (27.9%) | 5 (11.6%) | 8 (18.6%) | 6 (14.0%) | 16 (37.2%) | 6 (14.0%) | 9 (20.9%) |
| Autosomal Recessive Congenital Ichthyosis (ARCI) | 37 | 12 (32.4%) | 6 (16.2%) | 11 (29.7%) | 5 (13.5%) | 8 (21.6%) | 2 (5.4%) | 12 (32.4%) |
| X-linked ichthyosis | 20 | 3 (15.0%) | 4 (20.0%) | 2 (10.0%) | 1 (5.0%) | 7 (35.0%) | 0 (0.0%) | 6 (30.0%) |
| Epidermolytic ichthyosis | 13 | 1 (7.7%) | 1 (7.7%) | 0 (0.0%) | 3 (23.1%) | 8 (61.5%) | 1 (7.7%) | 2 (15.4%) |
| Netherton syndrome | 5 | 1 (20.0%) | 1 (20.0%) | 0 (0.0%) | 0 (0.0%) | 2 (40.0%) | 0 (0.0%) | 1 (20.0%) |
| **Statistical analysis of between-group effects** | - | χ^2^[4]=4.5, p=0.34 | χ^2^[4]=1.5, p=0.83 | χ^2^[4]=8.4, p=0.08 | χ^2^[4]=3.1, p=0.53 | χ^2^[4]=7.1, p=0.13 | χ^2^[4]=4.8, p=0.31 | χ^2^[4]=2.4, p=0.66 |

1. § Between-group effects analysed using chi-squared test, with significant Bonferroni-corrected p-values indicated by asterisks. [↑](#footnote-ref-1)
