# Supplementary material for: A mixed-methods online survey approach using retrospective self-reporting to characterise congenital ichthyoses across age groups
Source: Orphanet J Rare Dis. 2026 Apr 18;21:209. doi: 10.1186/s13023-026-04358-7 (PMC13224449; doi:10.1186/s13023-026-04358-7)
Supplement: Supplementary file 9 — Supplementary Material 9: Additional File 9. Factors contributing to changes in mobility across time periods [file 13023_2026_4358_MOESM9_ESM.docx]

**Additional File 9.** Factors contributing to changes in mobility across time periods

| **Type of ichthyosis** | **Number of participants reporting changing homeostatic condition** | **Number (%) of participants reporting factor as contributory towards changing mobility^[[1]](#footnote-1)^§** | | | | | | |
| --- | --- | --- | --- | --- | --- | --- | --- | --- |
|  |  | **Change in self-care** | **Change in personal circumstances** | **Change in living conditions** | **Change in medication or treatments** | **No obvious cause** | **Changes in medical or scientific advice** | **Other** |
| All types combined | 106 | 21 (19.8%) | 25 (23.6%) | 13 (12.3%) | 18 (17.0%) | 38 (35.8%) | 6 (5.7%) | 30 (28.3%) |
| Ichthyosis vulgaris | 45 | 8 (17.8%) | 11 (24.4%) | 7 (15.6%) | 7 (15.6%) | 11 (24.4%) | 3 (6.7%) | 14 (31.1%) |
| Autosomal Recessive Congenital Ichthyosis (ARCI) | 26 | 10 (38.5%) | 7 (26.9%) | 4 (15.4%) | 8 (30.8%) | 6 (23.1%) | 3 (11.5%) | 9 (34.6%) |
| X-linked ichthyosis | 18 | 1 (5.6%) | 1 (5.6%) | 1 (5.6%) | 0 (0.0%) | 12 (66.7%) | 0 (0.0%) | 5 (27.8%) |
| Epidermolytic ichthyosis | 13 | 1 (7.7%) | 5 (38.5%) | 1 (7.7%) | 3 (23.1%) | 7 (53.8%) | 0 (0.0%) | 1 (7.7%) |
| Netherton syndrome | 4 | 1 (25.0%) | 1 (25.0%) | 0 (0.0%) | 0 (0.0%) | 2 (50.0%) | 0 (0.0%) | 1 (25.0%) |
| **Statistical analysis of between-group effects** | - | χ^2^[4]=9.4, p=0.05 | χ^2^[4]=5.0, p=0.28 | χ^2^[4]=2.3, p=0.69 | χ^2^[4]=8.4, p=0.08 | χ^2^[4]=14.0, p=0.007* | χ^2^[4]=3.9, p=0.42 | χ^2^[4]=3.4, p=0.49 |

1. § Between-group effects analysed using chi-squared test, with significant Bonferroni-corrected p-values indicated by asterisks. [↑](#footnote-ref-1)
