# Supplementary material for: A mixed-methods online survey approach using retrospective self-reporting to characterise congenital ichthyoses across age groups
Source: Orphanet J Rare Dis. 2026 Apr 18;21:209. doi: 10.1186/s13023-026-04358-7 (PMC13224449; doi:10.1186/s13023-026-04358-7)
Supplement: Supplementary file 10 — Supplementary Material 10: Additional File 10. Factors contributing to changes in social relationships across time periods [file 13023_2026_4358_MOESM10_ESM.docx]

**Additional File 10.** Factors contributing to changes in social relationships across time periods

| **Type of ichthyosis** | **Number of participants reporting changing social relationships** | **Number (%) of participants reporting factor as contributory towards changing social relationships^[[1]](#footnote-1)^§** | | | | | | |
| --- | --- | --- | --- | --- | --- | --- | --- | --- |
|  |  | **Change in self-care** | **Change in personal circumstances** | **Change in living conditions** | **Change in medication or treatments** | **No obvious cause** | **Changes in medical or scientific advice** | **Other** |
| All types combined | 120 | 33 (27.5%) | 53 (44.2%) | 9 (7.5%) | 27 (22.5%) | 25 (20.8%) | 11 (9.2%) | 25 (20.8%) |
| Ichthyosis vulgaris | 46 | 12 (26.1%) | 18 (39.1%) | 5 (10.9%) | 9 (19.6%) | 7 (15.2%) | 5 (10.9%) | 12 (26.1%) |
| Autosomal Recessive Congenital Ichthyosis (ARCI) | 36 | 14 (38.9%) | 19 (52.8%) | 2 (5.6%) | 11 (30.6%) | 5 (13.9%) | 3 (8.3%) | 6 (16.7%) |
| X-linked ichthyosis | 18 | 2 (11.1%) | 9 (50.0%) | 1 (5.6%) | 2 (11.1%) | 5 (27.8%) | 1 (5.6%) | 5 (27.8%) |
| Epidermolytic ichthyosis | 16 | 3 (18.8%) | 5 (31.3%) | 0 (0.0%) | 3 (18.8%) | 8 (50.0%) | 2 (12.5%) | 1 (6.3%) |
| Netherton syndrome | 4 | 2 (50.0%) | 2 (50.0%) | 1 (25.0%) | 2 (50.0%) | 0 (0.0%) | 0 (0.0%) | 1 (25.0%) |
| **Statistical analysis of between-group effects** | - | χ^2^[4]=6.4, p=0.17 | χ^2^[4]=2.9, p=0.57 | χ^2^[4]=4.1, p=0.39 | χ^2^[4]=4.8, p=0.31 | χ^2^[4]=11.8, p=0.02 | χ^2^[4]=1.1, p=0.90 | χ^2^[4]=3.8, p=0.44 |

1. § Between-group effects analysed using chi-squared test, with significant Bonferroni-corrected p-values indicated by asterisks. [↑](#footnote-ref-1)
